# Tracking elemental changes in an ischemic stroke model with X-ray fluorescence imaging

**DOI:** 10.1038/s41598-020-74698-2

**Published:** 2020-10-20

**Authors:** M. J. Pushie, N. J. Sylvain, H. Hou, S. Caine, M. J. Hackett, M. E. Kelly

**Affiliations:** 1 Division of Neurosurgery, Department of Surgery, College of Medicine, University of Saskatchewan, Saskatoon, Canada; 2 College of Pharmacy and Nutrition, University of Saskatchewan, Saskatoon, Canada; 3 Department of Biomedical Sciences, Western College of Veterinary Medicine, University of Saskatchewan, Saskatoon, Canada; 4 Curtin Institute for Functional Molecules and Interfaces, School of Molecular and Life Sciences, Faculty of Science and Engineering, Curtin University, Kent Street, Bentley, Perth, WA 6102 Australia; 5 Curtin Health Innovation Research Institute, Curtin University, Bentley, WA 6102 Australia

**Keywords:** Biomarkers, Biomarkers, Blood-brain barrier, Cell death in the nervous system, Molecular neuroscience

## Abstract

Stroke is a leading cause of long-term disability in adults and a leading cause of death in developed nations. The cascade of cellular events and signalling that occur after cerebral ischemia are complex, however, analyzing global element markers of metabolic state affords the means to monitor stroke severity, status of injury, and recovery. These markers provide a multi-parameter method for assessing changes through the post-stroke time course. We employ synchrotron-based elemental mapping to follow elemental changes in the brain at 1 h, 1-, 2-, and 3-days, and at 1-, 2-, 3-, and 4-weeks post-stroke in a photothrombotic stroke model in mice. Our analysis reveals a highly consistent metabolic penumbra that can be readily identified based on the level of dysregulated potassium and other key elements. Maps of elemental distributions are also useful to demarcate events in the cellular response to the inflammatory cascade, including ion dysregulation, recruitment of cells to the lesion, and glial scar formation.

## Introduction

Cerebrovascular disease is the second most frequent cause of death worldwide, and the lifetime risk of suffering a stroke for the adult population (over 25 years old) is 25%^1^. Roughly 88% of all strokes are ischemic, as opposed to hemorrhagic stroke or transient ischemic attacks. During ischemic stroke, a volume of brain tissue is immediately starved of oxygen and nutrients supplied from the blood. Thrombolytics can be used acutely to treat large vessel occlusions, but have the potential to cause hemorrhagic transformation, while for patients with large vessel occlusion of the cerebral vessels, such as the middle cerebral artery, endovascular mechanical thrombectomy can be a highly efficacious alternative^2^. The most important patient characteristic determining post-stroke outcome is the amount of penumbra that can be salvaged by restoring normal blood flow^3–5^. The penumbra comprises the metabolically stressed tissue surrounding the infarct core and may be recoverable or may end up contributing to an increase in infarct size. Aside from histologic identification of the cellular characteristics of the penumbra, a systematic and quantitative characterization of this important metabolic region of tissue surrounding the core of the infarct provides useful insights into its potential to be rescued through intervention and treatment.

In the ischemic region insufficient ATP synthesis quickly leads to insufficient energy to maintain ATP-driven membrane transporters, which are essential for maintaining the electrochemical potential gradient needed for cell functioning. This loss of ion homeostasis, specifically in neurons, is an underlying driver of all downstream effects. Released glutamate binds to NMDA and AMPA receptors, promoting an influx of Na^+^, Ca^2+^, and water into these cells. In the brain Ca^2+^ overload actives the cells’ degradation machinery (proteases, lipases, and nucleases)^6,7^. Persistent glutamate levels leads to over excitation (excitotoxicity), creating a positive feedback signal for Ca^2+^ influx, which also activates the mitochondrial electron transport chain, increasing production of deleterious reactive oxygen (and nitrogen) species. Prior to cell death the cellular ion imbalance leads to an influx of water, leading to cytotoxic edema^8^. Increased blood brain barrier (BBB) permeability following stroke onset allows diffusion of components within the blood to permeate the brain parenchyma^9^.

We have highlighted the necessity for improved understanding and characterization of ions and elemental markers of cell dyshomeostasis in ischemic stroke^10^. Our team's first steps in this direction identified synchrotron-based X-ray fluorescence imaging (XFI) and infrared (IR)-based microscopy were exceptionally useful tools to supplement conventional methods such as histology and immunohistochemistry^9,10^. XFI affords the means to visualize the distribution of key elements in situ, which cannot otherwise be visualized without the introduction of reporter molecules. Fluorescent chelators with chemical specificity can be used to identify ions in tissue, however, these can only probe populations of ions that are labile and available to be coordinated by the chelator (i.e. not tightly bound to other biomolecules or sequestered in compartments inaccessible to the chelator). Moreover, common criticisms of chelation-based detection is the notorious promiscuity of such chelators to bind unintended ions as well as valid concerns that the chelator may only show particular populations of target ions or may demonstrate altered distribution due to cellular trafficking of the chelator. We recently demonstrated that Gaussian-based clustering with multiple elements from XFI maps can be a useful tool to differentiate metabolic differences in tissue sections from an ischemic stroke model at 24 h post-stroke^11^. The current time course study applies these methods to different time points post-stroke to track metabolically distinct regions from the early acute phase through to later time points.

## Results

### X-ray fluorescence energy detection and imaging

**Figure 1:**
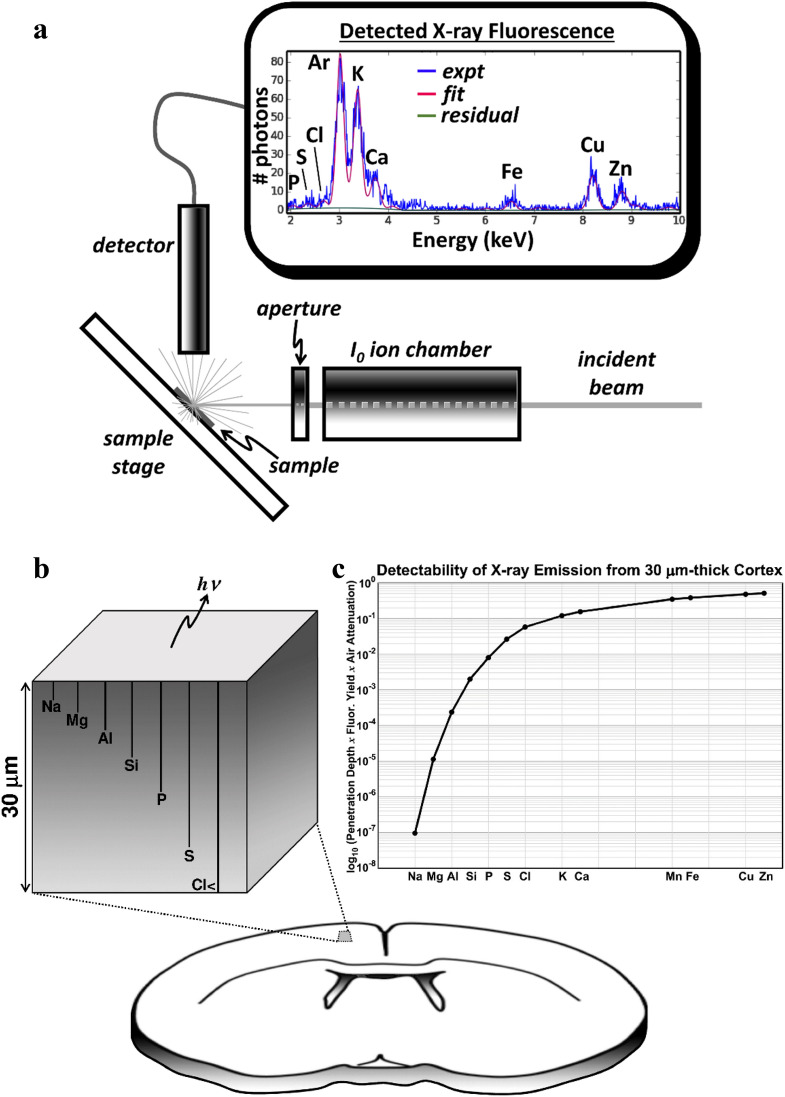
(**a**) Representative XFI setup. Measured X-ray fluorescence (shown in blue) is *fitted* with a linear combination of fluorescence peaks (in red) which are scaled to minimize the residual (green). (**b**) Depiction of the escape depth, from which 1/*e* of the elemental fluorescence escapes. The “detectability” of key elements (**c**) represents the depth from which 1/*e* of the signal at the indicated depth escapes the surface. After escaping the relatively dense matrix of the tissue the X-rays are also attenuated by the air gap. This attenuation is most significant for low energy emissions, such as from P and S, whereas lighter elements (e.g. Na and Mg) cannot be detected with these methods.

Synchrotron X-rays are used to induce an electronic excited state in a broad range of elements within a sample, and these elements then emit fluorescence which can be measured quantitatively for each element (e.g. the fluorescence spectrum shown in Fig. 1a). XFI detects the X-ray fluorescence spectrum point-by-point across the surface of a thin tissue section as described in Pushie et al.^12^ This is different from conventional medical imaging, such as X-ray or computed tomographic (CT) imaging, which are contrast-based techniques.

The incident X-ray beam, used to excite the tissue, is sufficiently high to penetrate the 30 μm-thick tissue, thereby exciting all elements with excitation energies below the incident energy (Fig. 1a). For most light elements their fluorescence emission energy is low enough that a significant proportion of the emitted fluorescence is attenuated before it can be detected. Figure 1b shows the approximate escape depth for a range of light elements (Na though Cl), showing the depth from which 1/*e* (~ 37%) of the X-ray fluorescent photons (*hν*) will escape from the surface of the sample. After escaping the tissue photons are further attenuated by the air-gap between the sample and the detector. Figure 1c shows the approximated “detectability” of a range of elements from a 30 μm-thick section of tissue using a typical sample-to-detector distance of 2.5 cm. “Detectability” in this instance assumes each element is at an equivalent concentration in the tissue and takes into account the escaping fluorescence from Fig. 1b, the air gap, and the fluorescence yield of each element (which increases with atomic number). Comparing equivalent concentrations of Na and Zn, for example, the detectability of Zn from within a section of tissue is near unity, whereas the detectability of Na is 7-orders of magnitude lower. This greatly limits the ability of the technique to probe the lightest elements and contributes to higher signal-to-noise for elements such as P and S in our data. Transition elements, like Fe, Cu, and Zn, have good detectability despite being of relatively low concentration in some tissue regions.

Our tissue preparation methods avoid the loss of mobile ions during perfusion or tissue fixation and preserve the in situ elemental levels and their gross distribution (see Methods)^13^. Brain tissue, for example, is high in K^+^, however, typical preparation methods (i.e. paraffin embedding and sectioning) result in nearly all of this element being lost from brain tissue (see Figure S1 in Supporting Information)^10,14^.

At present there are few studies employing XFI methods to study changes in ischemic or hemorrhagic stroke^9–11,15–22^. This paucity highlights the specialized nature of the team required to perform these types of studies. We have undertaken a comprehensive examination of post-stroke elemental changes in the photothrombotic (PT) mouse model, which is a highly reproducible model of focal thromboembolic ischemic stroke in the cortex. We employ conventional histological and immunohistochemical staining methods as well as quantitative XFI to follow the time course of the photothrombotic model, from 1-h up to 4-weeks post-stroke.

### General trends in the PT stroke model

**Figure 2:**
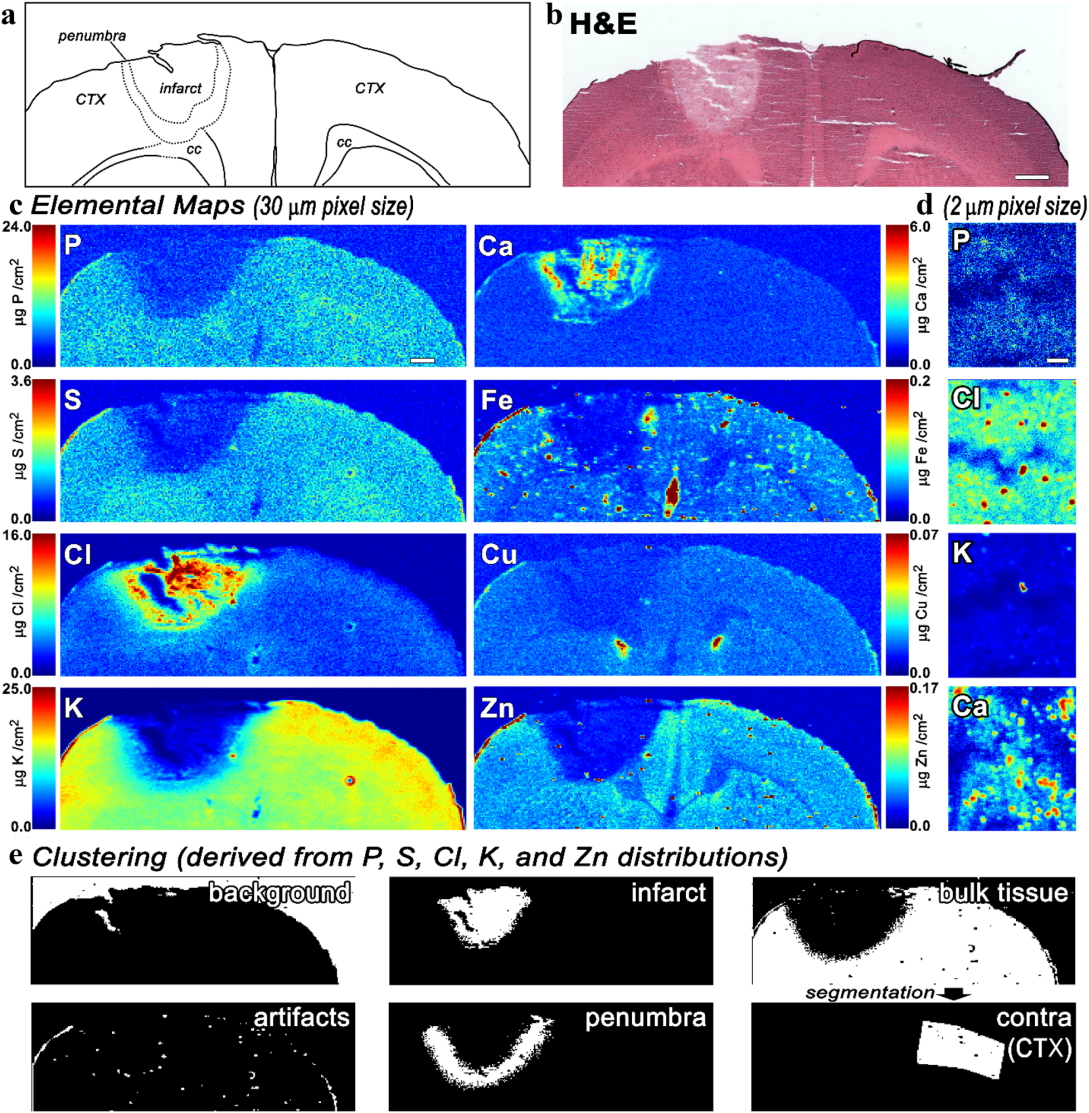
Representative trends for tissue changes occurring in the cortex of the PT model at 3-days post-stroke. (**a**) Schematic representation of relevant neuroanatomic features. (**b**) H&E stained section. (**c**) Elemental maps collected at 30 μm pixel size, (**d**) High-resolution XFI maps at 2 μm pixel size, and **e**) Defined regions of interest from clustering of XFI data. Scale bar in (**b** and **c**) = 500 microns, scale bar for (**d**) = 50 microns. CTX = cortex, cc = corpus callosum.

Hematoxylin and eosin (H&E) staining of the early post-stroke time points reveals reduced eosin staining of the neuropil in the core of the stroke lesion (infarct), with patchy eosin staining surrounding this area at early time points (Fig. 2a). Under higher magnification the cells within the stroke lesion are revealed to have shrunken, pyknotic nuclei with eosinophilic cytoplasm—a characteristic hallmark of degeneration following ischemic infarction^9,10^. Accurate mapping and quantification of the elemental distribution within tissue requires specimens be frozen with the shortest post-mortem interval possible and that no additional fixation or cryo-preservation steps are performed^10,14^. The snap-freeze method we employ preserves the in situ metabolic state of the tissue as closely as possible but results in tissue sections lacking the pristine appearance typical of embedded tissue (Fig. 2b).

The general trends of the early post-stroke interval are shown in Fig. 2 for all of the observable elements up to Zn, with the H&E and schematic depiction for reference. We note that the K and Ca distributions are subtly different from our previous Caine et al. paper^10^, as our previous work did not use software capable of adequately discriminating between overlapping fluorescence contributions from these two elements (e.g. Fig. 1a).

Within the stroke lesion we observe a reduction in P, S, K, Fe, Cu, and Zn, and a large increase in Cl and Ca. Higher-resolution imaging (2 μm pixel size, Fig. 2d) shows that there are few structural details within the core of the infarct, except for accumulations of Ca. After the tissue is sectioned, it is allowed to air-dry prior to XFI imaging. Within the first few minutes of this drying period, micro-crystals form within the infarct and it is these micro-crystals that are rich in Ca and lead to the mottled appearance of the Ca map. The core of the stroke lesion is comprised of necrotic tissue and we hypothesize that the presence of high Ca in this region results in spontaneous crystallization as the tissue dries of some of the highly elevated ionic species present (such as Ca^2+^, Asp^−^ and Glu^−^, as well as proteins and other small molecules). Supporting data on the micro-crystals is shown in Figure S2 in the Supporting Information accompanying this article. The micro-crystals demonstrate autofluorescence and are an artifact of tissue sectioning and drying and do not represent any inherent morphological or structural information in the stroke lesion (e.g. Fig. 2d). While some regions of the core of the infarct contain elevated hotspots of Cl that can be identified at higher resolution (right panel, Fig. 2c) the distribution of this element is generally more uniform at 30 μm pixel resolution and does not appear to be enriched in the micro-crystals. We note that early post-stroke time points, before the concentration of Ca has reached its maximum (i.e. 30 μm elemental map in Fig. 2) there appears to be elevated Ca within tissue surrounding the infarct that is not associated with concentrated microcrystals. Some of this difference in Ca distribution is loosely organized into a halo and may indicate a boundary region between the core of the stroke lesion and cells that have more recently undergone excitotoxic changes.

Dysregulation of Na^+^ has a central role in ischemic stroke, as its influx into cells is thought to be one of the primary contributors to cytotoxic edema. However, Na has a low fluorescence yield and the Na Kα X-ray fluorescence energy is too weak for detection with our methods (see Fig. 1c). Nevertheless, the concentration of Cl and Na in blood are both ~ 15× higher than average concentration in the brain and tracking changes in Cl levels is a reasonable proxy for assessing potential changes in Na once these ions begin to equilibrate with the core of the infarct.

### Defining regions of interest and identifying the ischemic penumbra

We have found that the early post-stroke region corresponding to the metabolic penumbra is not readily identifiable histologically, except for its shared border with the infarct core. To identify the entire penumbra requires the combined data from multiple elemental maps. These elemental maps are each indirect markers of (1) excitotoxic state of cells, (2) disruptions in membrane integrity, and (3) disrupted energy metabolism^9^. The statistical differences in the distribution and concentration of key elements associated with post-stroke changes affords the means to differentiate the regions of tissue in different metabolic states. Multi-dimensional clustering, using a Gaussian mixture model, uses the elemental distribution of P, S, Cl, K, and Zn to identify regions of interest (ROIs). As noted, the distribution of Ca at early post-stroke time points is an artifact of tissue drying and is therefore omitted from clustering. While the background signal of Fe within the stroke lesion is diminished, blood vessels and many of the inflammatory cells that later respond to the stroke lesion contain high Fe. The concentration and distribution of Fe is highly variable, we therefore omit Fe when defining clusters. Mn is generally at the detection limit of our technique and does not aid clustering, whereas Cu, although slightly reduced in the infarct, is generally at low levels overall in the tissue except for ependymal tissue surrounding the ventricles^23^ (see for e.g. Fig S3) and this tends to result in periventricular regions being assigned to a unique cluster. Generating clusters using the distribution of P, S, Cl, K, and Zn (Fig. 2e) provides an automated and reproducible method for defining ROIs based on the underlying metabolic state of the tissue^9^. The clustering approach we employ differentiates the core of the stroke lesion from bulk brain tissue and also identifies a region of tissue distinct from these surrounding the infarct (Fig. 2e). At early post-stroke time points, this additional region fits with the concept of an ischemic penumbra as the tissue within this border looks normal histologically.

With the ability to identify specific ROIs associated within the stroke lesion, the size of the infarct core and the surrounding penumbra can be tracked over time. The elemental levels within these regions serve as an indicator of the degree of dysregulation. As the PT stroke model largely targets the cortex, we isolated this region of the contralateral cortex to use as an internal reference (Fig. 2) for each time point and also compare this value to the same segmented ROI in shams. Performing clustering on shams using P, S, Cl, K, and Zn requires fewer clusters (3-to-4) overall and no ROIs are identified in the ipsilateral cortex of shams at any of the time points studied (including when the number of clusters is increased). When comparing the contralateral cortex of stroke brains to the cortex of age-matched shams, elemental levels showed few statistically significant differences overall—exceptions were 4 weeks S (*p* = 0.004), and 4 weeks Cl (*p* < 0.001). Statistical significance calculations are summarized in Supporting Information accompanying this article.

A key distinction with our identification of a metabolic penumbra is that at later time points the region surrounding the core, and even the core itself, correspond to different entities compared to the concept of the infarct and penumbra from earlier time points. As inflammatory cells migrate into the tissue surrounding the infarct and infarct core to aid glial scar formation they contribute to altered elemental composition and distribution in these regions. Furthermore, the concept of the penumbra as a region of potentially recoverable tissue no longer applies at later time points. We therefore differentiate the ROI surrounding the core as the penumbra at early post-stroke time points and as the peri-infarct zone (PIZ) at later time points (e.g. plots in Fig. 5). The core, however, is identified as “infarct core” throughout the time course herein. With these caveats in mind, the clustering still affords the means to differentiate metabolically distinct regions of tissue that are different from bulk brain tissue, as well as track changes in these regions over time.

### Tracking altered elemental levels post-stroke

**Figure 3:**
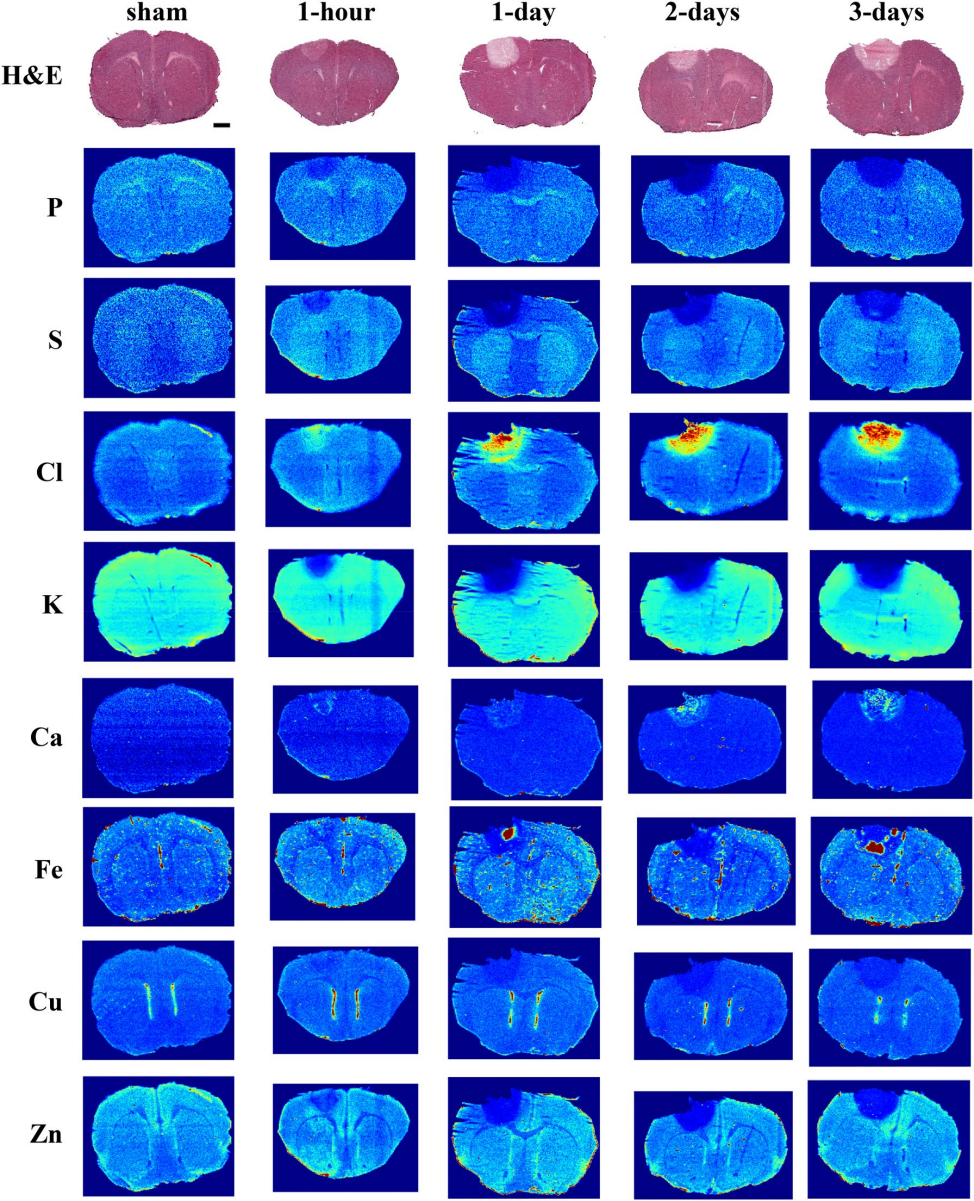
Representative XFI maps for early post-stroke time points from 1-h to 3-days post-stroke, with the adjacent H&E-stained tissue for each group. 1-day sham is shown for reference. Scale bar = 1 mm.

Looking at the changes that occur over early post-stroke time points, all elements have altered distributions in the stroke lesion as early as 1 h post-stroke (Fig. 3). Elemental levels in the infarct, with the exception of Ca and Fe, show statistically significant concentration differences from those in the contralateral cortex already at 1 h post-stroke (see Supporting Information), while Fe and Ca are showing changes by day 1. Although more subtle, the surrounding penumbra also shows significant changes in S, Cl, K and Zn at early post-stroke time points. The detectability of elements with XFI does not depend on the chemical form of the element, nor its partitioning within the tissue, therefore such changes cannot explain the altered levels we observe. We also note that elements with diminished concentrations within the stroke lesion are not significantly concentrated at the border, nor are elements with increased concentration depleted from the border. We hypothesize that these elemental changes are the result of equilibration across the BBB and this permeability appears to increase soon after stroke onset^9^, permitting mobile ions to freely diffuse. Both Cl and Ca, which represent pools of mobile ions (with some Ca sequestered separately in protein- and metabolite-bound adducts), are significantly elevated in blood^9^. These elements demonstrate a distinct pattern of elevated distribution within the core of the stroke lesion, as well as a second clearly-defined halo of elevated Cl and Ca around portions of the periphery. While the halo of elevated Cl and Ca are correlated, they do not colocalize with one another. These changes in elemental levels indicate that there is increased BBB permeability even by 1 h post-stroke. Cells of the endothelium are the first to experience ischemic conditions and swelling of these cells results in detachment from their basement membrane, compromising BBB integrity early after onset of ischemia.

In the PT stroke model, ischemia is generated at a focal location in the cortex through laser-induced photolysis within the blood vessels carrying the Rose Bengal dye. This generates localized thrombus formation at, and surrounding, the site of exposure to the laser, thereby occluding only these vessels. The occluded cerebral blood vessels themselves are not expected to significantly contribute to equilibration of extracellular components from the brain parenchyma and blood. We hypothesize that the exchange of elements is due to increased BBB permeability in collateral vessels surrounding the stroke lesion that are not thrombosed, thereby allowing the volume of tissue affected by the ischemic stroke to equilibrate with the blood volume.

**Figure 4:**
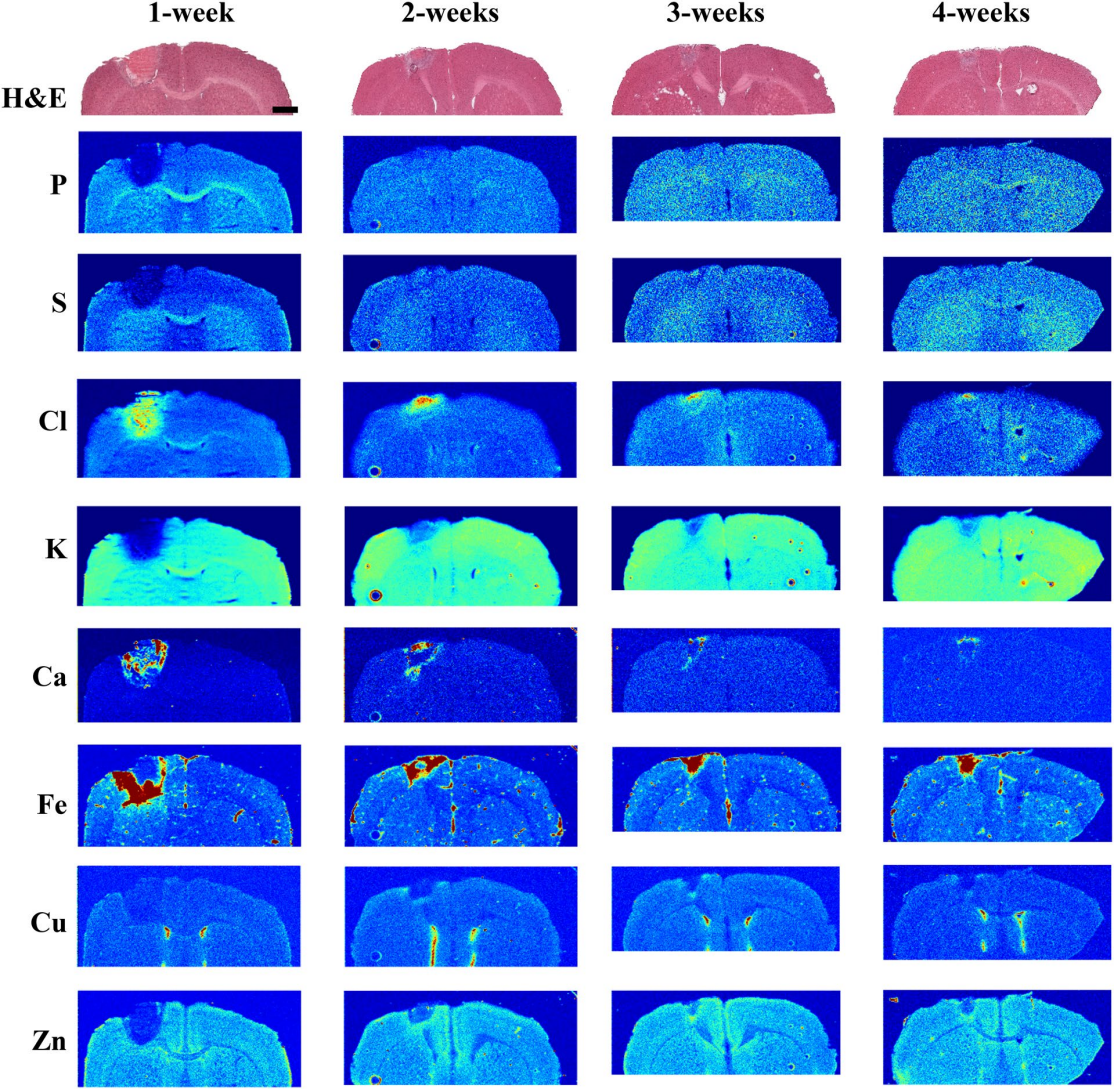
Representative XFI maps for late post-stroke time points from 1- to 4-weeks, with the adjacent H&E-stained tissue for each group. Shams are highly consistent across all time points and is omitted for simplicity (refer to Fig. 3 for representative images and to Fig. 5 for elemental quantification of shams). Scale bar = 1 mm.

The elemental changes evident at 1 h post-stroke are further amplified over the course of the first 3-days post-stroke. During this time the lesion size increases, as is evident from the region of altered elemental levels (K and Zn show this best), reaching its maximum size by day-3, with most of the largest changes in elemental levels (vs*.* sham) occurring by this time as well (Fig. 3). The Ca-enriched auto-fluorescent micro-crystals also reach maximal accumulation at day-3, whereas no micro-crystals are apparent at 1 h post-stroke, nor at later timepoints (1-week and beyond, Fig. 4).

**Figure 5:**
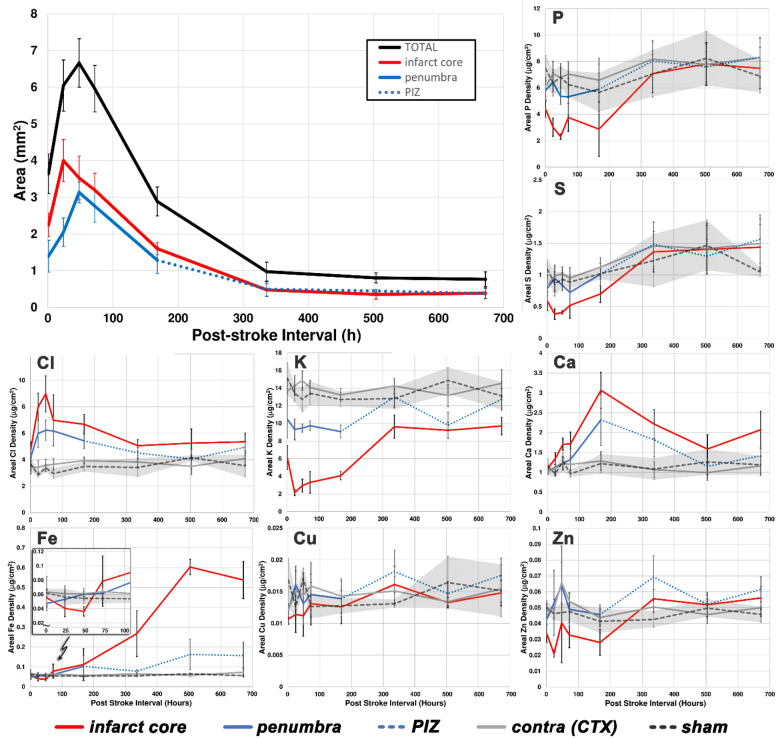
The area of the infarct and penumbra/PIZ are shown for the complete time course, averaged over all subjects. Quantified elemental levels from each ROI over the time course are shown for all elements. Error bars correspond to 95% confidence interval, with the CI for the sham group filled in grey for contrast in the elemental plots.

Following cellular depolarization after onset of ischemic stroke and later necrosis of the infarct core, much of the previously sequestered ions from this tissue are in the extra cellular space. With a permeable BBB any small molecules, including ions (sometimes refereed to as “free” ions), can flow down their concentration gradient. The bulk concentrations of elements between brain tissue and blood predict the flow of ions we observe. The concentration of Cl and Ca are ~ 10–15× and 13,000–30,000× more concentrated in blood than brain tissue (Na, although not observed herein is ~ 15× more concentrated in blood), whereas K and Zn are each ~ 30× more concentrated in the brain^24^. Magnesium, like Na, is not observable with XFI (see Fig. 1c), but is roughly equivalent between these two compartments, therefore major shifts in ion concentration are not anticipated.

### Altered elemental levels at late post-stroke time points

At later post-stroke time points the size of the lesion begins to shrink and is particularly notable when observing the distribution of Cl, Ca, and Fe (Fig. 4). Both Cl and Ca appear to be reduced from their post-stroke maximum over time, but the level of Fe continues to increase until 3-weeks post-stroke and remains elevated in all 3- and 4-week stroke animals (Fig. 5). This elevation in Fe is not due to a more compact distribution of Fe as the glial scar shrinks, and Fe levels remain significantly elevated after correcting for the reduced lesion size (not shown). Most elemental concentrations remain significantly altered from their normal levels within the affected region for the duration of the 4-week interval studied (Fig. 5).

Concomitantly with reduced stroke lesion size, we also observe indications of glial scar formation. Following onset of ischemia, resident microglia in the brain transform to an inflammatory phenotype which aids recruitment of further microglia, the brain’s resident immune cells, as well as circulating cells, including macrophages^25–27^. Transformed microglia undergo a morphological change from a ramified appearance to an ameboid state, which makes them nearly indistinguishable from macrophages^28,29^. Both macrophages and activated microglia are part of the inflammatory response following ischemic stroke. Both cell types contain the Fe-storage protein ferritin, which can store up to 4500 atoms of Fe per protein complex, and elevated Fe status is linked to activation of these cells^30,31^. Starting at day-2 there is a significant increase in Fe-rich hotspots in the periphery of the stroke lesion. These appear as roughly 1–2 pixels in size when imaged at 30 μm resolution and can be resolved to ~ 6 to 10 μm when imaged at 2 μm pixel size with a focused X-ray beam (see high resolution Fe map in Fig. S3 in Supporting Information). These Fe-rich hotspots accumulate around the periphery of the lesion and likely correspond to inflammatory cells.

**Figure 6:**
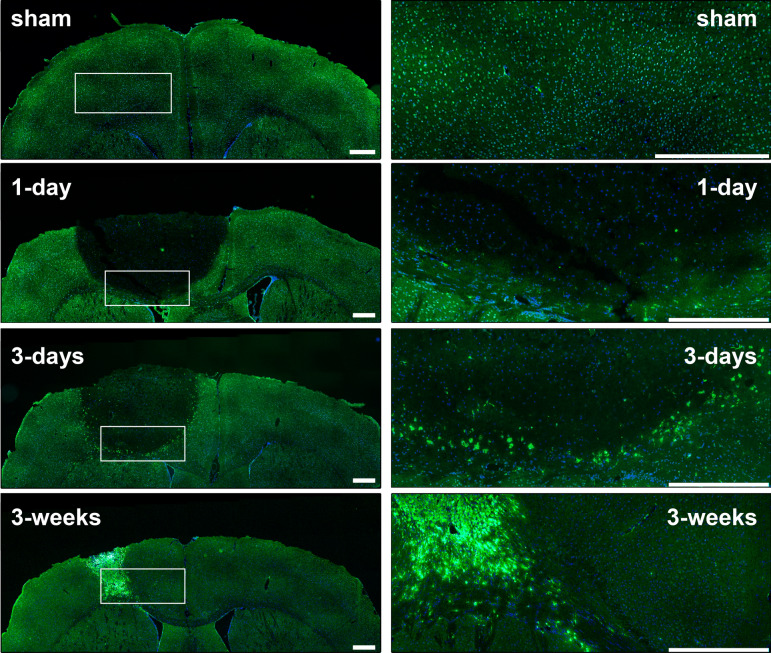
CD68 (green) and DAPI (blue) in sham brain relative to stroke brains at different time points post-stroke (1 day, 3 days and 3 weeks). The CD68+ cells can be seen at the infarct border starting after 1 day post-stroke. At later time points, CD68+ cells can be seen infiltrating the ischemic core to form a glial scar. Scale bar = 500 μm.

After a CNS injury like an ischemic stroke, a glial scar is formed by reactive astrocytes, NG2 glia and microglia forming a compact border around the site of injury, surrounding a lesion core composed of fibroblasts, pericytes, ependymal cells and phagocytic macrophage^32–34^. The glial scar serves to isolate and contain damaged tissue within the lesion, thus limiting further damage and contributing to neuroprotection^35^. In order to observe if these Fe-rich hotspots that accumulate around the periphery of the lesion may correspond to active inflammatory cells, we immunolabelled adjacent tissue sections with CD68, a marker of phagocytic activity in microglia and peripheral-derived macrophages^36–38^. At 3 days post-stroke, CD68+ cells can be seen in the periphery of the stroke lesion (Fig. 6) and this continues for several weeks post-stroke concomitant with the formation of a glial scar. At later time points, the lesion, largely containing CD68+ cells, begins to shrink, as evidenced by the reduction in area of affected tissue shown in Fig. 5 (seen in adjacent tissue sections, e.g. Fig. 6, to those used for XFI). Based on the observations of CD68 labelling and Fe distribution we conclude that the source of increased Fe signal is due to activation of CD68+ cells and their distribution within the glial scar.

Over the post-stroke duration studied the elemental levels in the infarct core begin to normalize at later time points for P, S, and Cu, while other elements remain significantly altered from those in the contralateral cortex (see Supporting Information). The altered elemental levels at later post-stroke time points is the result of the infiltration of functional glial cells and macrophages into this region of tissue, but the trend toward normal elemental levels does not necessarily signify functional recovery.

Analysing the radial distribution of elements from the centre of the stroke lesion out to a distance of 1.5 mm reveals the cross section of altered elemental distribution. For example, at 3-days post-stroke, the radial distribution for K and Zn (two of the elements with the most distinct post-stroke change) are shown in Figure S4 (see Supporting Information). While there is significant difference between the core of the infarct (closer to the origin) and normal tissue at the periphery (i.e*.* beyond 1 mm), the intermediate region is much more variable. Across the early post-stroke time points, this region contains the metabolic penumbra. The peaks in the derivative plots (overlaid with the radial distributions in Fig. S4) indicate points of maximal change.

### Tracking post-stroke lesion size

The stroke lesion of the PT model reaches its maximum size by 2-days post-stroke (taking into account infarct core + penumbra) and begins to shrink thereafter. Based on the ROI defined by Gaussian-based clustering of the XFI data the total area of the penumbra reaches its maximum at 24-h post-stroke, after which the areas corresponding to the penumbra diminish while the infarct core continues to grow.

While it is not possible to track the post-stroke changes longitudinally in the same subject with our imaging techniques we can nevertheless identify general trends from the aggregated data. The area of the infarct core grows into the penumbra between 1- and 24-h post-stroke, reaching a maximum at 24-h. The area of the infarct core changes minimally between days 1 through 3, while the penumbra continues to grow steadily until reaching its maximum by day 2. Both of these regions are trending toward a smaller area from post-stroke day-3 onward. The data does not provide any measure of the water content surrounding the stroke lesion and this may be an additional dynamic factor influencing the apparent areal changes between infarct core and penumbra. It is likely that hydrostatic edema contributes to some of the observed changes in lesion area.

## Discussion

We observe two distinct phases of metabolic response to the induced ischemic insult. Initially, ion dyshomeostasis results in significant changes in ion levels in the affected tissue. This results in initial loss of P, S, K, Fe, Cu, and Zn, and increased Cl and Ca, which are already markedly changed from their normal levels by 1-h post-stroke. By 24-h post-stroke the changes in elemental levels are further amplified. Iron-rich macrophages and reactive microglia accumulate in the periphery of the stroke lesion (Fig. 6) between 2-to-3-days post-stroke, and this accumulation continues into the chronic post-stroke phase. In addition to Fe, these invading cells bring their own compliment of ions and other elements necessary for their normal function, but differs from the elemental levels that would otherwise be present in healthy brain tissue.

Loss of perfusion quickly leads to depletion of ATP and failure of ATP-dependent Na^+^/K^+^ pumps. Increased extracellular K^+^ is a generally accepted consequence of metabolic failure in ischemic tissue, however, our imaging findings demonstrate that the entire stroke lesion contains decreased K overall, particularly within the infarct core and increasing outward through the penumbra back to baseline levels (healthy tissue). The central dogma of ischemic stroke pathophysiology is also linked to an influx of Ca^2+^ into dysregulated cells. The detected levels of Ca (and to a lesser extent Cl) observed with XFI is significantly higher than is present in the cortex of healthy tissue and appears to represent Ca^2+^ derived from an external source (e.g. blood). The significant elevation in Ca within the ischemic core and peri-infarct tissues implies there could be a greater Ca^2+^ burden to overcome than previously thought for any hope of functional recovery.

Clustering^11,22^ affords the means to identify metabolically distinct areas of tissue, allowing the differentiation of the infarct core from bulk brain tissue and an intermediate region surrounding the infarct core which we identify as the metabolic penumbra at early post-stroke time points or the PIZ at later time points. Clustering provides the means to track changes in element concentrations as well as the size in the regions over time. The present time course reveals significant reductions in P, S, and K within the infarct core have already occurred by 1-h post-stroke (*p* values are 0.0002, < 0.0001, and < 0.0001, respectively) and although the levels of P and S begin to approach normal levels by 1-week the K level in the core of the lesion remains below normal levels throughout (all *p* values are < 0.0001, see Supporting Information accompanying this article). There is also increased Cl relative to normal cortex levels within the infarct core as early as 1 h post-stroke and remains elevated throughout the rest of the time course. The Ca level within the infarct core is not significantly elevated above the normal reference for cortex at 1 h post-stroke (*p* value 0.9814), but becomes significant by post-stroke day-3 and remains elevated (*p* values for day-3 and beyond are < 0.0001) at all time points. Elemental levels within the region identified as the penumbra or peri-infarct zone are intermediate between the core and normal tissue levels. The responding inflammatory cells alter the average elemental composition of the stroke lesion, therefore elemental levels at 1-week post-stroke and beyond represent a combination of the elemental levels in these cells as well as the surrounding tissue.

Heeding the aphorism that “time is brain”, the photothrombotic model demonstrates a that the critical window for saving a large proportion of the penumbra is within the first 24-h, while after this time the penumbra expands to encompass previously spared tissue. Importantly, the large change in elemental levels, such as ions like K^+^ and Ca^2+^, that are already evident in the stroke lesion by 1-h post-stroke, is an early indication of significant dysfunction, likely making this tissue unrecoverable. The total area of tissue that is affected in the acute phase (which includes infarct core and penumbra) reaches a maximum at 2-days post-stroke. This capability to differentiate the metabolic states in tissue will be particularly useful to track stroke severity in the presence of exacerbating stroke risk factors, as well as the quantifying the efficacy of stroke treatments in animal models.

## Methods

### Animal models

A total of 104 Male BALB/c mice, housed in the University Saskatchewan’s Large Animal Services Unit, were maintained in a 12 h light/dark cycle, with ad libitum access to chow and water. The photothrombotic (PT) stroke model was employed following established methods^10,39^. Eleven week-old mice were anesthetized using isoflurane (3% induction, 1.5–2% maintenance in 40% O_2_ and 60% N_2_O, Baxter Corp., Toronto ON, CA). The fur above the skull was shaved and the head secured in a stereotactic frame. The skull was exposed with an incision in the midline of the scalp. The area of skull overlying the primary somatosensory cortex was identified for alignment of the laser (S1FL, − 1 to − 2.5 mm midline-to-lateral; + 1 to − 0.5 mm anterior-to-posterior of bregma, right hemisphere). An intraperitoneal injection of the photoreactive dye, Rose Bengal (100 mg/kg, Sigma, USA), was administered and the dye was allowed to circulate for 5 min. A sterile mask was applied surrounding the site to minimize scatter of the incident laser light and the S1FL was irradiated for 20 min with a green laser (532 nm) to photoactivate the Rose Bengal, with the source positioned 3 cm above the skull. Laser power was in the 23–24.5 mW range, confirmed prior to each use using a C-Series Optical Power Meter (Thorlabs, Inc.). Post-stroke time zero coincides with laser shut off. Sham mice underwent the same procedures described above but did not receive exposure to the laser. Marcaine (1.67 mg/mL, 18 μL/15 g) was applied to the open wound before suturing and animals recovered in single animal cages post-surgery. Animals were kept for the following post-stroke durations before euthanasia: 1-h, 1-, 2-, 3-days, and 1-, 2-, 3-, and 4-weeks. Animals with euthanasia time points longer than 1 week had their sutures removed at 1 week and were group housed until their pre-determined euthanasia time point. Each post-stroke time point had *n* = 8 PT animals, while the sham group for each time point had *n* = 5. The smaller number of shams was justified based on the good reproducibility of elemental data acquired at each time point for this group.

Animals were euthanized by heavily anaesthetizing with isoflurane, followed immediately by removing the heads with a decapitator. To preserve biochemical speciation and distribution of labile ions, the heads were immediately submerged in liquid N_2_ to rapidly freeze the tissue^14^. Heads were maintained at ca. − 20 °C while the fur, skin, muscle, and bone were chiseled away from the frozen brain. Brains were sectioned coronally using a cryomicrotome, at an operating temperature of − 18 °C, starting + 0.25 mm anterior to bregma. Tissue sections for histology and immunohistochemistry were cut at 14 μm and collected onto glass microscope slides and stored at − 80 °C until staining, while adjacent tissues for XFI were cut at 30 μm-thick onto metal-free Nunc Thermanox coverslips (Thermo Fisher Scientific Inc.) allowed to air-dry at ambient temperature before analysis. Frozen brains that were too badly fractured during extraction from the skull were excluded from analysis and animals that were euthanized for ethical reasons prior to reaching their designated post-stroke time point were also excluded.

All animal work was conducted with approval from the University of Saskatchewan’s Animal research Ethics Board and carried out in accordance with the Canadian Council on Animal Care guidelines for humane animal use.

### Histology and immunohistochemistry

Adjacent tissue sections on glass slides were fixed in 4% buffered paraformaldehyde, washed with 0.1 M phosphate buffered saline (PBS), and then stained with hematoxylin and eosin (H&E) as previously described (Caine et al., 2016). For immunohistochemistry labelling of macrophage, adjacent tissues were first fixed in 4% buffered paraformaldehyde, washed with PBS and then incubated in a blocking solution for 1 h (1% bovine serum albumin, 0.02% secondary host serum, 0.001% Triton X, PBS). Tissues were incubated in anti-CD68 (Abcam, Cambridge, UK) at 1:400 in blocking solution overnight at 4 °C, washed in PBS and then incubated at room temperature for 2 h in donkey anti-rabbit Alexa Fluor 488 secondary antibody (Invitrogen) at 1:200 with 1:10 000 DAPI (Molecular Probes) in blocking solution, followed by washing in PBS. To block tissue autofluorescence, tissues were incubated in 0.05% Sudan Black B (Sigma) in 70% ethanol for 5mins, followed by washing in distilled water, and cover slipped using Prolong gold antifade mountant (Invitrogen).

### Microscopy

Hematoxylin and eosin (H&E) tissues were imaged using a Leica Aperio virtual microscope using a 20× objective lens and images were extracted using Aperio ImageScope (Leica). Immunohistochemistry fluorescent tissues were imaged using a 20× objective lens on a Leica DM6000 B microscope, fitted with a motorized stage and a Leica DFC365FX camera, which is part of the offline equipment at the BioXAS-Imaging beamline at the Canadian Light Source Inc. synchrotron (Canada).

### XFI data acquisition

XFI was carried out as previously described^11^ at the Stanford Synchrotron Radiation Lightsource (SSRL) on beamlines 10-2 and 2-3, with the SPEAR3 storage ring operating in top-up mode at 3 GeV and 500 mA. Beamline 10-2 is equipped with a 33-pole 1.45-T wiggler, using a Si(111) double-crystal monochromator (ϕ = 90° orientation) and a Rh-coated mirror for focusing. A microfocussed beam, approximately 35 μm × 35 μm, was achieved using an aperture downstream of the I_0_ ion chamber. Samples were mounted at 45° to the incident beam and raster scanned using Newport IMS Series stages (Irvine CA, USA) in 30 μm steps, providing an oversampling pixel size of 30 μm, using a dwell time of 200 ms per point. Beamline 2-3 is equipped with a 1.3 T bend magnet. Data was collecting using a Si(111) double-crystal monochromator and a Kirkpatrick-Baez mirror system to achieve a beam size of ~ 2 μm × 2 μm. Newport stages were used with a 2 μm step size, and a dwell time of 200 ms per point. A schematic of the beamline setup for both beamlines is shown in Fig. 1a.

Imaging on both beamlines used an incident energy that was set below the Br K-edge, at 13,450 eV. A silicon-drift Vortex detector was positioned 45° to the sample normal (90° to the incident beam). On beamline 10-2 a detector was fitted with a collimator to reduce total counts from scattered X-rays in the sample area. Both beamlines utilize the Xpress3 signal processing system (Quantum Detectors, UK) and the full multi-channel array spectrum for each point in the XFI maps were saved for off-line processing.

### XFI data processing

Multi-channel array spectra for each pixel in the XFI map were processed using the MicroAnalysis Toolkit SMAK^40^, which fits the total X-ray fluorescence emission spectrum using a series of emission lines for each element. This method of deconvolution allows contributions from overlapping fluorescence lines to be separated, and their individual contributions to the spectrum quantified. For example, the K Kα and Kβ emission lines overlap with Ca Kα (and vice versa), which can lead to incorrect quantification of elements. This peak fitting approach yields more accurate elemental quantification and improves signal-to-noise overall. The fitted X-ray fluorescence intensities were converted to aerial concentrations (μg/cm^2^) in SMAK using reference standards deposited on 6.3 μm-thick mylar film. These standards include: K and Cl (KCl, 98.8 μg/cm^2^), Ca (CaF_2_, 56.8 μg/cm^2^), P (GaP, 47.0 μg/cm^2^), Fe (Fe, 56.0 μg/cm^2^), S and Cu (CuS_x_, Cu = 74.9 μg/cm^2^, S = 21.0 μg/cm^2^), and Zn (ZnTe, 45.8 μg/cm^2^) (Micromatter, Vancouver, CA). Elemental maps were extracted for plotting in ImageJ^41^ to ensure elemental maps across all time points were plotted with at the same intensity (grayscale intensities were displayed using the Jet lookup table in ImageJ).

Radial plots of elemental distributions (Fig. S4), centred on the core of the infarct, were generated using the Radial Profile Extended^42^ plug-in for ImageJ^42^. Briefly, the coordinates of the core of the stroke lesion were determined such that the affected tissue was uniformly contained within the bounds of a given radius, the size of which varies with post-stroke interval. A 30° wedge was sampled from the core of the lesion toward the contralateral cortex, without including the corpus callosum, and the radial profiles of elemental concentrations were averaged. To identify points of significant change across the stroke lesion, the first derivative of the averaged radial distribution was calculated using 5-pixel smoothing to minimize noise.

XFI data from P, S, Cl, K, and Zn for each pixel were collectively analyzed using expectation maximization (EM), a Gaussian mixture-based soft clustering method similar to that described previously for similar synchrotron imaging data^43^. Each ROI was randomly seeded and optimized using K-means clustering during the initial analysis pass. The refined K-means clusters were then used to seed subsequent clustering using EM. The log likelihood was calculated for each EM run and used as an objective measure to identify completion.

Multi-dimensional clustering was performed using the data mining and visualization package Orange^44^. Elemental maps were imported from SMAK into Orange and the elemental maps for P, S, Cl, K, and Zn were employed for clustering analysis using the Gaussian mixture model algorithm from Scikit-Learn^45^. An initial round of K-means clustering is performed, which is seeded randomly, and the results of the K-means clustering is used to seed subsequent rounds of a Gaussian-mixture-model fit of the data. We employ 5 clusters for the majority of samples which are identifiable upon inspection as off-sample background, normal brain tissue, the core of the stroke lesion, a region of tissue surrounding the core of the stroke lesion, and artifacts arising from the edges of the sample. In some instances, additional clusters were required when there were folds in the tissue (which makes the intensity of elemental signals artificially high) or in cases were trace element-containing dust contaminated tissue sections. In all instances, the additional clusters could be accounted for due to spurious sources of variability in the underlying data and are therefore omitted from analysis.

### Statistical analysis

Elemental concentrations in the infarct and penumbra/PIZ were compared to contralateral cortex concentrations at each time point. In general, contralateral elemental levels in the cortex (the primary region affected in the PT stroke model) were not significantly different from age-matched sham cortex levels (see SI for full summary). Additionally, infarct and penumbra/PIZ lesion sizes were compared between time points. Statistical analysis was performed using the Kruskal–Wallis nonparametric analysis of variance method^46^, followed by a post-hoc test of multiple comparisons using the Conover-Iman method^47,48^. A *p* value ≤ 0.01 was employed to test for significance (Table S1). Elemental levels shown in Fig. 5 correspond to a 95% confidence interval.

## Supplementary information

Supplementary information
