# Supplementary material for: Tracking elemental changes in an ischemic stroke model with X-ray fluorescence imaging
Source: Sci Rep. 2020 Oct 20;10:17868. doi: 10.1038/s41598-020-74698-2 (PMC7575585; doi:10.1038/s41598-020-74698-2)
Supplement: Supplementary file 1 — Supplementary information [file 41598_2020_74698_MOESM1_ESM.docx]

**Supporting Information for**

**Tracking Elemental Changes in an Ischemic Stroke Model with X-ray Fluorescence Imaging**

Pushie MJ,*^1^* Sylvain NJ,*^1^* Hou H,*^1^* Caine S,*^2,3^* Hackett MJ,*^4,5^* Kelly ME*^1*^*

*^1^Division of Neurosurgery**, Department of Surgery, College of Medicine, University of Saskatchewan, Canada.*

*^2^* *College of Pharmacy and Nutrition, University of Saskatchewan, Canada.*

*^3^ Department of Biomedical Sciences, Western College of Veterinary Medicine, University of Saskatchewan, Canada.*

*^4^Curtin Institute for Functional Molecules and Interfaces, School of Molecular and Life Sciences, Faculty of Science & Engineering, Curtin University, Kent Street, Bentley, Perth, Western Australia 6102, Australia*

*^5^Curtin Health Innovation Research Institute, Curtin University, Bentley, Western Australia 6102, Australia.*

* Corresponding Author: m.kelly@usask.ca

**Figure S1.** Conventional tissue preparation steps (*e.g.* soaking brains in buffered paraformaldehyde, followed by paraffin embedding). The section was collected after being floated on a water bath. The concentration of labile ions is significantly reduced in the tissue - most notably are the maps of Cl and K, as both elements persist in the tissue as freely diffusible ions. The lines that are most evident in the Zn map are due to wrinkles in the tissue specimen. Colour intensities are matched to those in Figure 3. Scale bar = 450 μm.


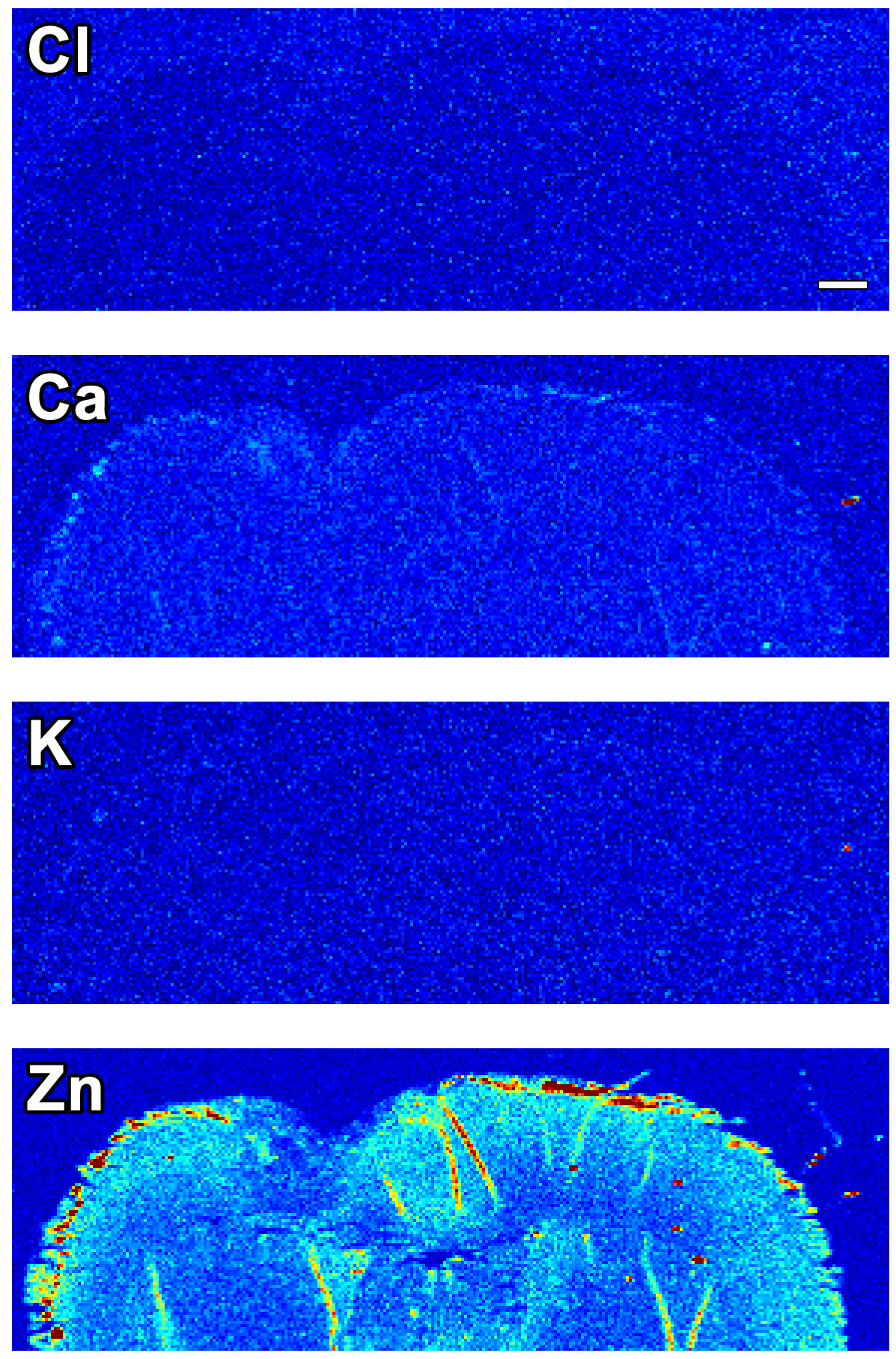


**Figure S2.** Representative images of a 3-day post-stroke tissue section. Timed images (top panels) are visible images from a light microscope with a polarization filter. These show that as the frozen tissue sections are warmed to room temperature and dry, microcrystals form at nucleation sites. These microcrystals appear as dark spots (indicated by arrows). Elemental maps show these spots are high in calcium. The microcrystals are auto-fluorescent and must be quenched using Sudan black prior to fluorescent immunohistochemistry (not shown). Scale bar in visible images = 50 μm. Scale bar in schematic image and XFI maps = 1 mm.


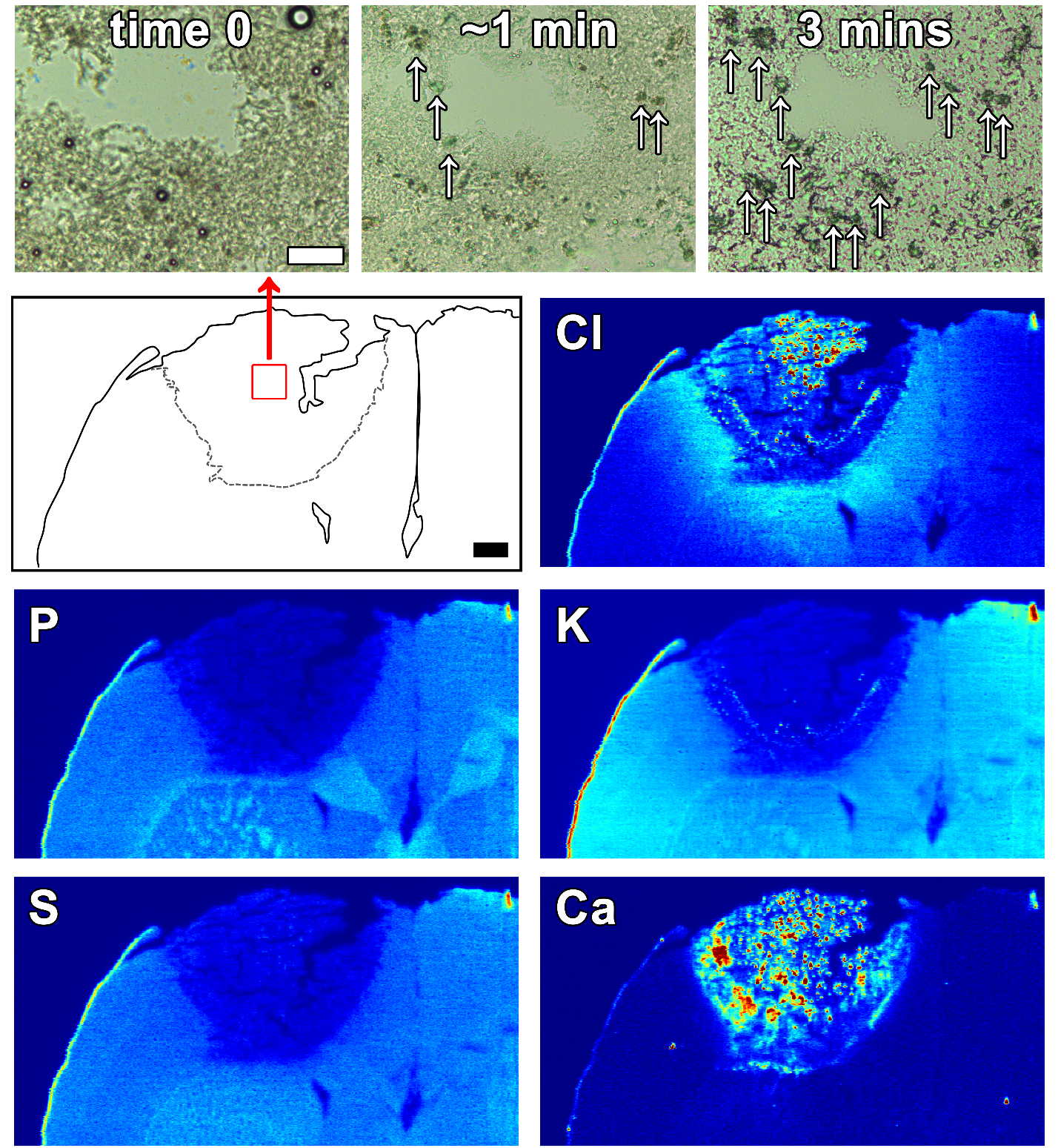


**Figure S3:** High spatial resolution XFI data (2 μm pixel size). (a) Schematic representation of the 3-week post-stroke tissue. (b) Fe, Cu, and Zn maps demonstrating the distribution of high Fe-containing hotspots (corresponding to CD68+ cells), which migrate along the corpus callosum toward the stroke lesion during recruitment. (c) Enlarged Fe map, highlighting the size of the Fe hotspots. Scale bar in schematic image and Fe, Cu, and Zn maps = 100 μm. Scale bar in zoomed-in Fe map = 20 μm.

**
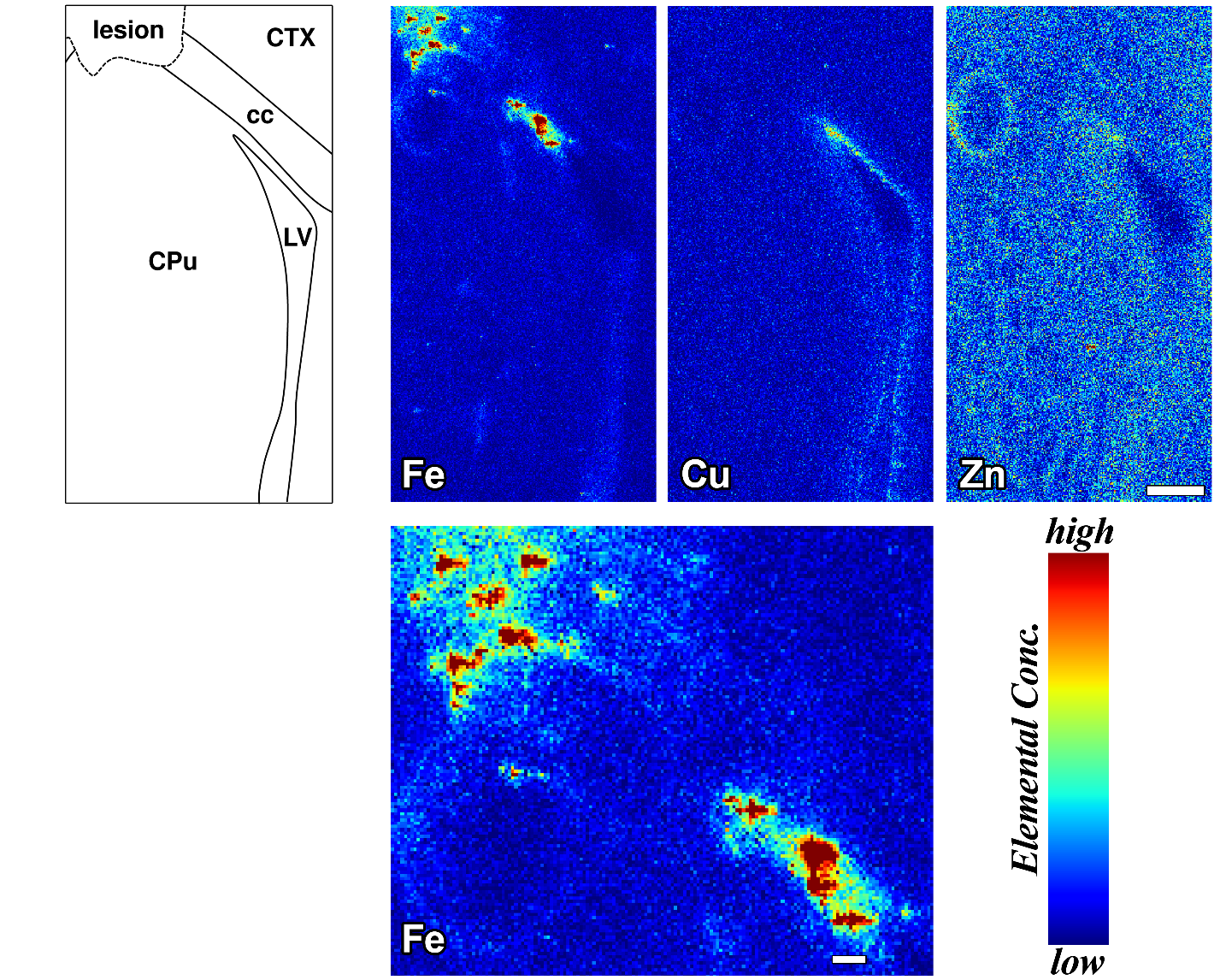
**

**a b**

**c**

**Figure S4**. Representative radial distribution plots of K and Zn are shown at 3-days post-stroke, with the infarct core at the origin, showing a gradual return toward normal tissue levels. The derivative for each radial distribution is shown, with maxima at 25 and 36 pixels from the centre of the region (corresponding to a distance of 0.75 mm and 1.08 mm, roughly corresponding to the transitions from infarct core to penumbra and penumbra to bulk tissue).


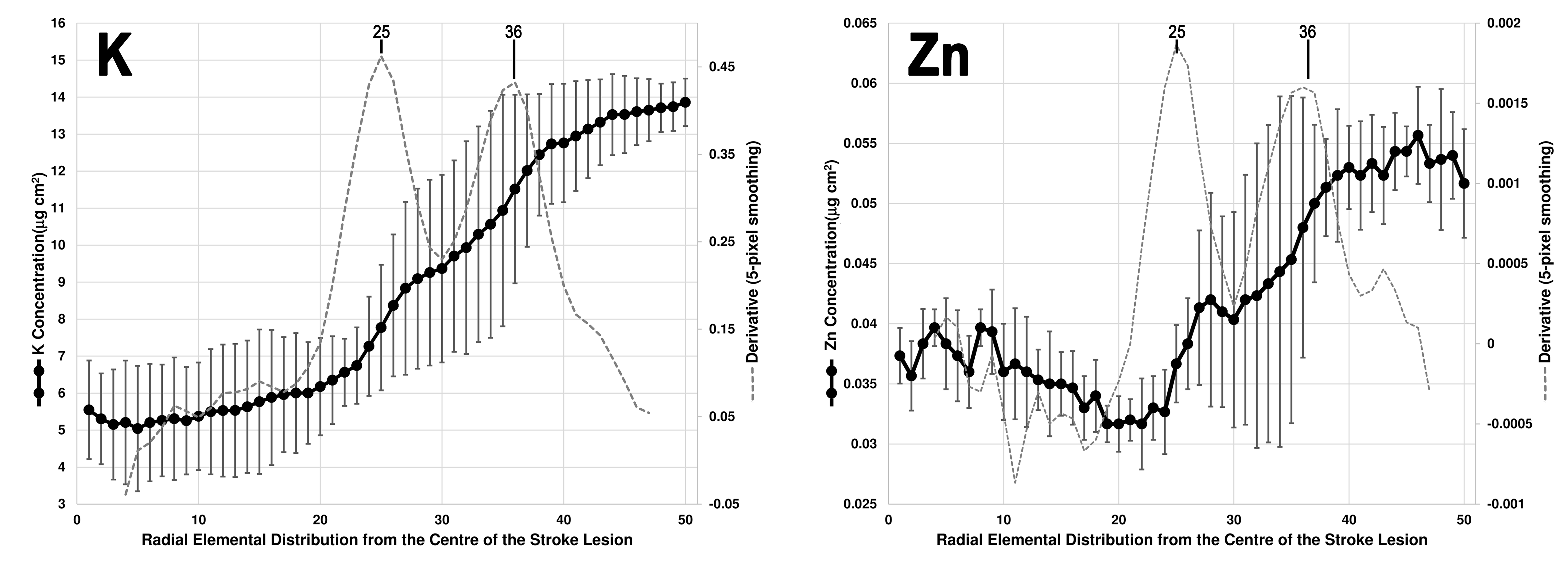

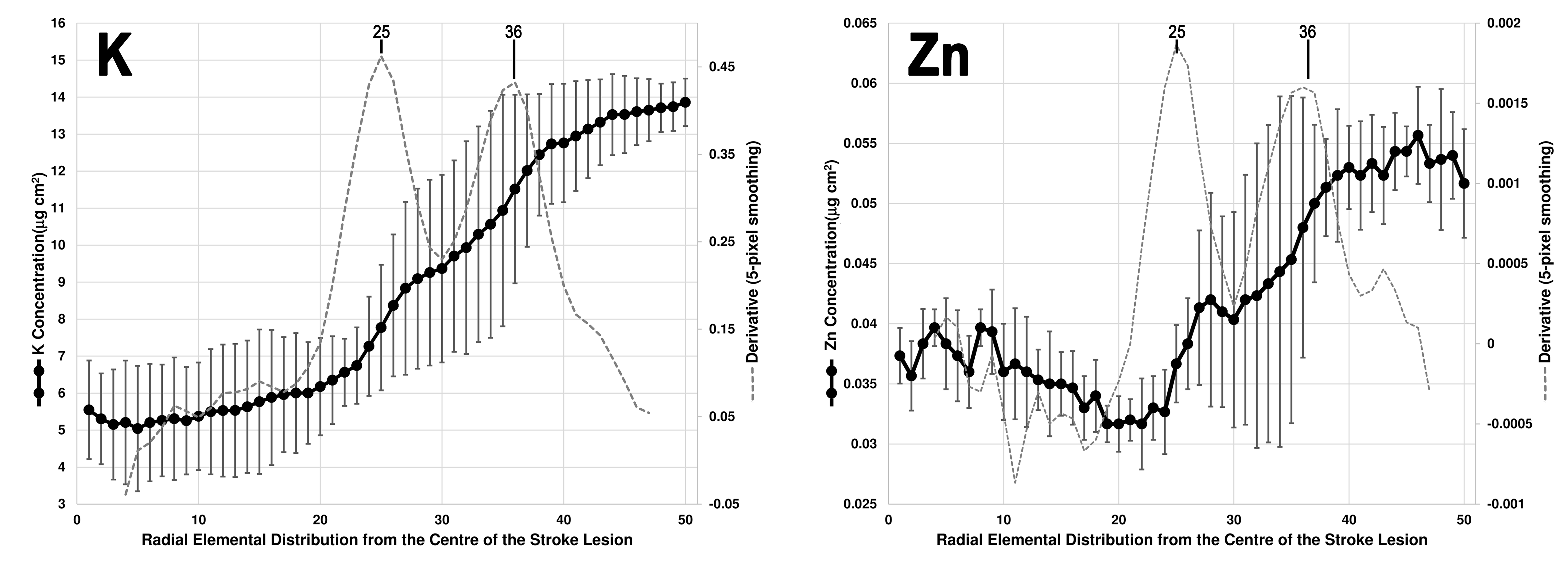


Distance from the center of the stroke lesion (mm)

Distance from the center of the stroke lesion (mm)

**Table S1.** p-values from Conover-Iman pairwise comparisons following Kruskal-Wallis test.

Statistically significant differences (below 0.01) are highlighted in blue.

***Infarct***

| **P** | 1h | 1d | 2d | 3d | 1wk | 2wk | 3wk | 4wk | *contra* |
| --- | --- | --- | --- | --- | --- | --- | --- | --- | --- |
| 1h | − | 0.5336 | 0.3053 | 0.8806 | 0.1584 | 0.0052 | 0.0006 | 0.0007 | 0.0002 |
| 1d |  | − | 0.5916 | 0.5979 | 0.3296 | 0.0002 | <0.0001 | <0.0001 | <0.0001 |
| 2d |  |  | − | 0.3303 | 0.6961 | 0.0002 | <0.0001 | <0.0001 | <0.0001 |
| 3d |  |  |  | − | 0.1628 | 0.0013 | <0.0001 | <0.0001 | <0.0001 |
| 1wk |  |  |  |  | − | <0.0001 | <0.0001 | <0.0001 | <0.0001 |
| 2wk |  |  |  |  |  | − | 0.5900 | 0.6833 | 0.7856 |
| 3wk |  |  |  |  |  |  | − | 0.8724 | 0.6461 |
| 4wk |  |  |  |  |  |  |  | − | 0.7868 |
|  |  |  |  |  |  |  |  |  | − |

***Penumbra / Peri-infarct Zone***

| **P** | 1h | 1d | 2d | 3d | 1wk | 2wk | 3wk | 4wk | *contra* |
| --- | --- | --- | --- | --- | --- | --- | --- | --- | --- |
| 1h | − | 0.7318 | 0.1917 | 0.8476 | 0.6357 | 0.0938 | 0.1784 | 0.0634 | 0.1870 |
| 1d |  | − | 0.0806 | 0.5575 | 0.3640 | 0.1192 | 0.2383 | 0.0739 | 0.2422 |
| 2d |  |  | − | 0.2336 | 0.3498 | 0.0044 | 0.0094 | 0.0021 | 0.0056 |
| 3d |  |  |  | − | 0.7650 | 0.0485 | 0.1007 | 0.0273 | 0.0847 |
| 1wk |  |  |  |  | − | 0.0239 | 0.0519 | 0.0117 | 0.0349 |
| 2wk |  |  |  |  |  | − | 0.6670 | 0.9787 | 0.3520 |
| 3wk |  |  |  |  |  |  | − | 0.6099 | 0.6793 |
| 4wk |  |  |  |  |  |  |  | − | 0.2384 |
|  |  |  |  |  |  |  |  |  | − |

***Infarct***

| **S** | 1h | 1d | 2d | 3d | 1wk | 2wk | 3wk | 4wk | *contra* |
| --- | --- | --- | --- | --- | --- | --- | --- | --- | --- |
| 1h | − | 0.3652 | 0.4303 | 0.7458 | 0.8092 | <0.0001 | 0.0001 | <0.0001 | <0.0001 |
| 1d |  | − | 0.9757 | 0.5409 | 0.2721 | <0.0001 | <0.0001 | <0.0001 | <0.0001 |
| 2d |  |  | − | 0.6054 | 0.3292 | <0.0001 | <0.0001 | <0.0001 | <0.0001 |
| 3d |  |  |  | − | 0.5792 | <0.0001 | <0.0001 | <0.0001 | <0.0001 |
| 1wk |  |  |  |  | − | 0.0003 | 0.0007 | <0.0001 | 0.0003 |
| 2wk |  |  |  |  |  | − | 0.6385 | 0.9897 | 0.2174 |
| 3wk |  |  |  |  |  |  | − | 0.5890 | 0.4908 |
| 4wk |  |  |  |  |  |  |  | − | 0.2161 |
|  |  |  |  |  |  |  |  |  |  |

***Penumbra / Peri-infarct Zone***

| **S** | 1h | 1d | 2d | 3d | 1wk | 2wk | 3wk | 4wk | *contra* |
| --- | --- | --- | --- | --- | --- | --- | --- | --- | --- |
| 1h | − | 0.2565 | 0.9454 | 0.8815 | 0.6371 | 0.0004 | 0.0066 | <0.0001 | 0.0044 |
| 1d |  | − | 0.2572 | 0.1759 | 0.5764 | 0.0059 | 0.0650 | 0.0007 | 0.0822 |
| 2d |  |  | − | 0.9453 | 0.6084 | 0.0007 | 0.0085 | <0.0001 | 0.0076 |
| 3d |  |  |  | − | 0.5290 | 0.0001 | 0.0030 | <0.0001 | 0.0011 |
| 1wk |  |  |  |  | − | 0.0037 | 0.0340 | 0.0006 | 0.0442 |
| 2wk |  |  |  |  |  | − | 0.3764 | 0.6834 | 0.0494 |
| 3wk |  |  |  |  |  |  | − | 0.1795 | 0.4236 |
| 4wk |  |  |  |  |  |  |  | − | 0.0048 |
|  |  |  |  |  |  |  |  |  |  |

***Infarct***

| **Cl** | 1h | 1d | 2d | 3d | 1wk | 2wk | 3wk | 4wk | *contra* |
| --- | --- | --- | --- | --- | --- | --- | --- | --- | --- |
| 1h | − | 0.0027 | 0.0026 | 0.0986 | 0.0273 | 0.5259 | 0.7100 | 0.3454 | 0.0111 |
| 1d |  | − | 0.6790 | 0.1260 | 0.4639 | 0.0117 | 0.0031 | 0.0104 | <0.0001 |
| 2d |  |  | − | 0.0864 | 0.3059 | 0.0099 | 0.0033 | 0.0099 | <0.0001 |
| 3d |  |  |  | − | 0.4803 | 0.2852 | 0.1506 | 0.3597 | <0.0001 |
| 1wk |  |  |  |  | − | 0.0913 | 0.0396 | 0.1079 | <0.0001 |
| 2wk |  |  |  |  |  | − | 0.7590 | 0.7878 | 0.0003 |
| 3wk |  |  |  |  |  |  | − | 0.5303 | 0.0004 |
| 4wk |  |  |  |  |  |  |  | − | <0.0001 |
|  |  |  |  |  |  |  |  |  |  |

***Penumbra / Peri-infarct Zone***

| **Cl** | 1h | 1d | 2d | 3d | 1wk | 2wk | 3wk | 4wk | *contra* |
| --- | --- | --- | --- | --- | --- | --- | --- | --- | --- |
| 1h | − | 0.0006 | 0.0007 | 0.0009 | 0.0021 | 0.2011 | 0.5711 | 0.0022 | 0.2973 |
| 1d |  | − | 0.6606 | 0.9878 | 0.8910 | 0.0319 | <0.0001 | 0.6549 | <0.0001 |
| 2d |  |  | − | 0.6603 | 0.5964 | 0.0230 | 0.0001 | 0.4128 | <0.0001 |
| 3d |  |  |  | − | 0.9057 | 0.0390 | 0.0001 | 0.6788 | <0.0001 |
| 1wk |  |  |  |  | − | 0.0617 | 0.0003 | 0.7863 | <0.0001 |
| 2wk |  |  |  |  |  | − | 0.0669 | 0.0797 | 0.0069 |
| 3wk |  |  |  |  |  |  | − | 0.0003 | 0.7741 |
| 4wk |  |  |  |  |  |  |  | − | <0.0001 |
|  |  |  |  |  |  |  |  |  |  |

***Infarct***

| **K** | 1h | 1d | 2d | 3d | 1wk | 2wk | 3wk | 4wk | *contra* |
| --- | --- | --- | --- | --- | --- | --- | --- | --- | --- |
| 1h | − | 0.0242 | 0.1821 | 0.9303 | 0.6377 | 0.0661 | 0.0361 | 0.0039 | <0.0001 |
| 1d |  | − | 0.5202 | 0.0233 | 0.0715 | 0.0001 | <0.0001 | <0.0001 | <0.0001 |
| 2d |  |  | − | 0.1923 | 0.3516 | 0.0046 | 0.0019 | 0.0002 | <0.0001 |
| 3d |  |  |  | − | 0.6858 | 0.0469 | 0.0255 | 0.0018 | <0.0001 |
| 1wk |  |  |  |  | − | 0.0236 | 0.0104 | 0.0008 | <0.0001 |
| 2wk |  |  |  |  |  | − | 0.9489 | 0.4661 | <0.0001 |
| 3wk |  |  |  |  |  |  | − | 0.4534 | <0.0001 |
| 4wk |  |  |  |  |  |  |  | − | <0.0001 |
|  |  |  |  |  |  |  |  |  |  |

***Penumbra / Peri-infarct Zone***

| **K** | 1h | 1d | 2d | 3d | 1wk | 2wk | 3wk | 4wk | *contra* |
| --- | --- | --- | --- | --- | --- | --- | --- | --- | --- |
| 1h | − | 0.3096 | 0.2634 | 0.3764 | 0.1431 | 0.0120 | 0.7127 | 0.0138 | <0.0001 |
| 1d |  | − | 0.7743 | 0.9721 | 0.6027 | 0.0004 | 0.4952 | 0.0003 | <0.0001 |
| 2d |  |  | − | 0.7680 | 0.8774 | 0.0012 | 0.3995 | 0.0013 | <0.0001 |
| 3d |  |  |  | − | 0.6146 | 0.0014 | 0.5650 | 0.0014 | <0.0001 |
| 1wk |  |  |  |  | − | 0.0001 | 0.2432 | <0.0001 | <0.0001 |
| 2wk |  |  |  |  |  | − | 0.0031 | 0.7399 | 0.1185 |
| 3wk |  |  |  |  |  |  | − | 0.0029 | <0.0001 |
| 4wk |  |  |  |  |  |  |  | − | 0.0177 |
|  |  |  |  |  |  |  |  |  |  |

***Infarct***

| **Ca** | 1h | 1d | 2d | 3d | 1wk | 2wk | 3wk | 4wk | *contra* |
| --- | --- | --- | --- | --- | --- | --- | --- | --- | --- |
| 1h | − | 0.1111 | 0.0015 | 0.0019 | <0.0001 | <0.0001 | 0.0006 | <0.0001 | 0.9814 |
| 1d |  | − | 0.0481 | 0.0760 | 0.0002 | 0.0038 | 0.0328 | 0.0017 | 0.0217 |
| 2d |  |  | − | 0.6907 | 0.1356 | 0.4692 | 0.9390 | 0.4908 | <0.0001 |
| 3d |  |  |  | − | 0.0392 | 0.2218 | 0.7193 | 0.2105 | <0.0001 |
| 1wk |  |  |  |  | − | 0.4102 | 0.0836 | 0.3059 | <0.0001 |
| 2wk |  |  |  |  |  | − | 0.3774 | 0.9110 | <0.0001 |
| 3wk |  |  |  |  |  |  | − | 0.3833 | <0.0001 |
| 4wk |  |  |  |  |  |  |  | − | <0.0001 |
|  |  |  |  |  |  |  |  |  |  |

***Penumbra / Peri-infarct Zone***

| **Ca** | 1h | 1d | 2d | 3d | 1wk | 2wk | 3wk | 4wk | *contra* |
| --- | --- | --- | --- | --- | --- | --- | --- | --- | --- |
| 1h | − | 0.5483 | 0.1576 | 0.0800 | 0.0022 | 0.0017 | 0.2385 | 0.0245 | 0.5387 |
| 1d |  | − | 0.3618 | 0.2231 | 0.0080 | 0.0066 | 0.5254 | 0.0848 | 0.8668 |
| 2d |  |  | − | 0.8567 | 0.1033 | 0.1059 | 0.7597 | 0.5684 | 0.2068 |
| 3d |  |  |  | − | 0.1083 | 0.1094 | 0.5954 | 0.6662 | 0.0765 |
| 1wk |  |  |  |  | − | 0.9196 | 0.0443 | 0.1873 | 0.0008 |
| 2wk |  |  |  |  |  | − | 0.0427 | 0.1941 | 0.0004 |
| 3wk |  |  |  |  |  |  | − | 0.3318 | 0.3337 |
| 4wk |  |  |  |  |  |  |  | − | 0.0100 |
|  |  |  |  |  |  |  |  |  |  |

***Infarct***

| **Fe** | 1h | 1d | 2d | 3d | 1wk | 2wk | 3wk | 4wk | *contra* |
| --- | --- | --- | --- | --- | --- | --- | --- | --- | --- |
| 1h | − | 0.0040 | 0.0089 | 0.8015 | 0.1940 | 0.0003 | <0.0001 | <0.0001 | 0.3666 |
| 1d |  | − | 0.8214 | 0.0012 | <0.0001 | <0.0001 | <0.0001 | <0.0001 | <0.0001 |
| 2d |  |  | − | 0.0038 | 0.0004 | <0.0001 | <0.0001 | <0.0001 | 0.0002 |
| 3d |  |  |  | − | 0.2639 | 0.0004 | <0.0001 | <0.0001 | 0.5267 |
| 1wk |  |  |  |  | − | 0.0215 | 0.0012 | 0.0019 | 0.3975 |
| 2wk |  |  |  |  |  | − | 0.3692 | 0.5184 | 0.0001 |
| 3wk |  |  |  |  |  |  | − | 0.7580 | <0.0001 |
| 4wk |  |  |  |  |  |  |  | − | <0.0001 |
|  |  |  |  |  |  |  |  |  |  |

***Penumbra / Peri-infarct Zone***

| **Fe** | 1h | 1d | 2d | 3d | 1wk | 2wk | 3wk | 4wk | *contra* |
| --- | --- | --- | --- | --- | --- | --- | --- | --- | --- |
| 1h | − | 0.1200 | 0.0200 | 0.0077 | <0.0001 | <0.0001 | <0.0001 | <0.0001 | 0.0009 |
| 1d |  | − | 0.3036 | 0.1948 | 0.0021 | 0.0005 | <0.0001 | <0.0001 | 0.0717 |
| 2d |  |  | − | 0.8934 | 0.0527 | 0.0141 | 0.0062 | 0.0009 | 0.7985 |
| 3d |  |  |  | − | 0.0468 | 0.0114 | 0.0039 | 0.0004 | 0.9136 |
| 1wk |  |  |  |  | − | 0.4891 | 0.4563 | 0.1988 | 0.0193 |
| 2wk |  |  |  |  |  | − | 0.9689 | 0.6661 | 0.0039 |
| 3wk |  |  |  |  |  |  | − | 0.5818 | 0.0004 |
| 4wk |  |  |  |  |  |  |  | − | <0.0001 |
|  |  |  |  |  |  |  |  |  |  |

***Infarct***

| **Cu** | 1h | 1d | 2d | 3d | 1wk | 2wk | 3wk | 4wk | *contra* |
| --- | --- | --- | --- | --- | --- | --- | --- | --- | --- |
| 1h | − | 0.4602 | 0.4646 | 0.1228 | 0.6839 | 0.0004 | 0.0275 | 0.0016 | 0.0017 |
| 1d |  | − | 0.1634 | 0.3619 | 0.7985 | 0.0017 | 0.0958 | 0.0071 | 0.0081 |
| 2d |  |  | − | 0.0395 | 0.2918 | 0.0002 | 0.0090 | 0.0007 | 0.0008 |
| 3d |  |  |  | − | 0.3020 | 0.0239 | 0.4329 | 0.0920 | 0.1707 |
| 1wk |  |  |  |  | − | 0.0029 | 0.0911 | 0.0112 | 0.0174 |
| 2wk |  |  |  |  |  | − | 0.1505 | 0.4082 | 0.1007 |
| 3wk |  |  |  |  |  |  | − | 0.4380 | 0.7864 |
| 4wk |  |  |  |  |  |  |  | − | 0.4190 |
|  |  |  |  |  |  |  |  |  |  |

***Penumbra / Peri-infarct Zone***

| **Cu** | 1h | 1d | 2d | 3d | 1wk | 2wk | 3wk | 4wk | *Contra* |
| --- | --- | --- | --- | --- | --- | --- | --- | --- | --- |
| 1h | − | 0.0238 | 0.2534 | 0.2505 | 0.8621 | 0.0022 | 0.0759 | 0.0014 | 0.1177 |
| 1d |  | − | 0.0012 | 0.2126 | 0.0381 | 0.2442 | 0.6837 | 0.2610 | 0.1377 |
| 2d |  |  | − | 0.0243 | 0.1935 | <0.0001 | 0.0056 | <0.0001 | 0.0056 |
| 3d |  |  |  | − | 0.3365 | 0.0249 | 0.4497 | 0.0199 | 0.8496 |
| 1wk |  |  |  |  | − | 0.0037 | 0.1102 | 0.0026 | 0.1817 |
| 2wk |  |  |  |  |  | − | 0.1471 | 0.8598 | 0.0076 |
| 3wk |  |  |  |  |  |  | − | 0.1518 | 0.4303 |
| 4wk |  |  |  |  |  |  |  | − | 0.0028 |
|  |  |  |  |  |  |  |  |  |  |

***Infarct***

| **Zn** | 1h | 1d | 2d | 3d | 1wk | 2wk | 3wk | 4wk | *contra* |
| --- | --- | --- | --- | --- | --- | --- | --- | --- | --- |
| 1h | − | 0.0260 | 0.2191 | 0.7210 | 0.3806 | 0.0611 | 0.1059 | 0.0395 | <0.0001 |
| 1d |  | − | 0.5411 | 0.0492 | 0.2433 | 0.0002 | <0.0001 | <0.0001 | <0.0001 |
| 2d |  |  | − | 0.3337 | 0.6827 | 0.0063 | 0.0090 | 0.0028 | <0.0001 |
| 3d |  |  |  | − | 0.5635 | 0.0260 | 0.0397 | 0.0113 | <0.0001 |
| 1wk |  |  |  |  | − | 0.0114 | 0.0165 | 0.0048 | <0.0001 |
| 2wk |  |  |  |  |  | − | 0.5757 | 0.8155 | <0.0001 |
| 3wk |  |  |  |  |  |  | − | 0.6728 | <0.0001 |
| 4wk |  |  |  |  |  |  |  | − | <0.0001 |
|  |  |  |  |  |  |  |  |  |  |

***Penumbra / Peri-infarct Zone***

| **Zn** | 1h | 1d | 2d | 3d | 1wk | 2wk | 3wk | 4wk | *contra* |
| --- | --- | --- | --- | --- | --- | --- | --- | --- | --- |
| 1h | − | 0.9415 | 0.8077 | 0.3659 | 0.6106 | 0.0159 | 0.3872 | 0.0221 | <0.0001 |
| 1d |  | − | 0.7534 | 0.3136 | 0.5562 | 0.0103 | 0.3256 | 0.0131 | <0.0001 |
| 2d |  |  | − | 0.5734 | 0.8118 | 0.0569 | 0.6237 | 0.0887 | <0.0001 |
| 3d |  |  |  | − | 0.7573 | 0.1436 | 0.8973 | 0.2301 | <0.0001 |
| 1wk |  |  |  |  | − | 0.0974 | 0.8290 | 0.1534 | <0.0001 |
| 2wk |  |  |  |  |  | − | 0.0845 | 0.6488 | <0.0001 |
| 3wk |  |  |  |  |  |  | − | 0.1344 | <0.0001 |
| 4wk |  |  |  |  |  |  |  | − | <0.0001 |
|  |  |  |  |  |  |  |  |  |  |

***Infarct***

| **Size** | 1h | 1d | 2d | 3d | 1wk | 2wk | 3wk | 4wk |  |
| --- | --- | --- | --- | --- | --- | --- | --- | --- | --- |
| 1h | − | 0.0073 | 0.2130 | 0.9177 | 0.4992 | 0.0031 | <0.0001 | 0.2017 |  |
| 1d |  | − | 0.2017 | 0.0066 | 0.0017 | <0.0001 | <0.0001 | <0.0001 |  |
| 2d |  |  | − | 0.2304 | 0.0725 | 0.0002 | <0.0001 | <0.0001 |  |
| 3d |  |  |  | − | 0.4249 | 0.0017 | <0.0001 | <0.0001 |  |
| 1wk |  |  |  |  | − | 0.0236 | 0.0012 | 0.0019 |  |
| 2wk |  |  |  |  |  | − | 0.3639 | 0.5371 |  |
| 3wk |  |  |  |  |  |  | − | 0.6970 |  |
| 4wk |  |  |  |  |  |  |  | − |  |
|  |  |  |  |  |  |  |  |  |  |

***Penumbra / Peri-infarct Zone***

| **Size** | 1h | 1d | 2d | 3d | 1wk | 2wk | 3wk | 4wk |  |
| --- | --- | --- | --- | --- | --- | --- | --- | --- | --- |
| 1h | − | 0.0358 | 0.0001 | 0.0016 | 0.9529 | 0.0013 | 0.0013 | <0.0001 |  |
| 1d |  | − | 0.0164 | 0.2009 | 0.0411 | <0.0001 | <0.0001 | <0.0001 |  |
| 2d |  |  | − | 0.1824 | 0.0001 | <0.0001 | <0.0001 | <0.0001 |  |
| 3d |  |  |  | − | 0.0019 | <0.0001 | <0.0001 | <0.0001 |  |
| 1wk |  |  |  |  | − | 0.0011 | 0.0011 | <0.0001 |  |
| 2wk |  |  |  |  |  | − | >0.9999 | 0.4771 |  |
| 3wk |  |  |  |  |  |  | − | 0.4771 |  |
| 4wk |  |  |  |  |  |  |  | − |  |
|  |  |  |  |  |  |  |  |  |  |

***Contra vs Sham***

| **P** | 1h | 1d | 2d | 3d | 1wk | 2wk | 3wk | 4wk | *Sham* |
| --- | --- | --- | --- | --- | --- | --- | --- | --- | --- |
| 1h | − | 0.2428 | 0.9699 | 0.2831 | 0.7075 | 0.0745 | 0.1697 | 0.0576 | 0.3361 |
| 1d |  | − | 0.2708 | 0.9543 | 0.3759 | 0.3722 | 0.7414 | 0.3271 | 0.6127 |
| 2d |  |  | − | 0.3029 | 0.7391 | 0.0814 | 0.1834 | 0.0636 | 0.3621 |
| 3d |  |  |  | − | 0.4213 | 0.3601 | 0.7090 | 0.3184 | 0.6868 |
| 1wk |  |  |  |  | − | 0.1022 | 0.2475 | 0.0745 | 0.5216 |
| 2wk |  |  |  |  |  | − | 0.5745 | 0.9790 | 0.1295 |
| 3wk |  |  |  |  |  |  | − | 0.5475 | 0.3759 |
| 4wk |  |  |  |  |  |  |  | − | 0.0718 |
|  |  |  |  |  |  |  |  |  | − |

***Contra vs Sham***

| **S** | 1h | 1d | 2d | 3d | 1wk | 2wk | 3wk | 4wk | *Sham* |
| --- | --- | --- | --- | --- | --- | --- | --- | --- | --- |
| 1h | − | 0.0470 | 0.0893 | 0.1658 | 0.0130 | 0.0007 | 0.0110 | 0.0002 | 0.0373 |
| 1d |  | − | 0.9157 | 0.5159 | 0.5227 | 0.0980 | 0.4798 | 0.0690 | 0.6580 |
| 2d |  |  | − | 0.6342 | 0.4974 | 0.1106 | 0.4597 | 0.0861 | 0.8074 |
| 3d |  |  |  | − | 0.2102 | 0.0246 | 0.1870 | 0.0132 | 0.6914 |
| 1wk |  |  |  |  | − | 0.3248 | 0.9483 | 0.2890 | 0.2279 |
| 2wk |  |  |  |  |  | − | 0.3573 | 0.9749 | 0.0143 |
| 3wk |  |  |  |  |  |  | − | 0.3225 | 0.1973 |
| 4wk |  |  |  |  |  |  |  | − | 0.0037 |
|  |  |  |  |  |  |  |  |  | − |

***Contra vs Sham***

| **Cl** | 1h | 1d | 2d | 3d | 1wk | 2wk | 3wk | 4wk | *Sham* |
| --- | --- | --- | --- | --- | --- | --- | --- | --- | --- |
| 1h | − | 0.4948 | 0.9902 | 0.2549 | 0.1066 | 0.0925 | 0.4046 | 0.0031 | 0.4459 |
| 1d |  | − | 0.5535 | 0.5999 | 0.2843 | 0.2517 | 0.8271 | 0.0111 | 0.9283 |
| 2d |  |  | − | 0.3228 | 0.1578 | 0.1409 | 0.4627 | 0.0109 | 0.5352 |
| 3d |  |  |  | − | 0.5779 | 0.5286 | 0.7863 | 0.0519 | 0.4546 |
| 1wk |  |  |  |  | − | 0.9436 | 0.4288 | 0.1977 | 0.1641 |
| 2wk |  |  |  |  |  | − | 0.3890 | 0.2252 | 0.1379 |
| 3wk |  |  |  |  |  |  | − | 0.0341 | 0.7289 |
| 4wk |  |  |  |  |  |  |  | − | 0.0009 |
|  |  |  |  |  |  |  |  |  | − |

***Contra vs Sham***

| **K** | 1h | 1d | 2d | 3d | 1wk | 2wk | 3wk | 4wk | *Sham* |
| --- | --- | --- | --- | --- | --- | --- | --- | --- | --- |
| 1h | − | 0.7479 | 0.3035 | 0.9192 | 0.1253 | 0.7163 | 0.1472 | 0.6820 | 0.1851 |
| 1d |  | − | 0.1544 | 0.6631 | 0.1565 | 0.9423 | 0.1865 | 0.9238 | 0.2276 |
| 2d |  |  | − | 0.3493 | 0.0145 | 0.1604 | 0.0178 | 0.1279 | 0.0154 |
| 3d |  |  |  | − | 0.1017 | 0.6383 | 0.1203 | 0.5985 | 0.1449 |
| 1wk |  |  |  |  | − | 0.2122 | 0.9277 | 0.1707 | 0.4908 |
| 2wk |  |  |  |  |  | − | 0.2467 | 0.9900 | 0.3368 |
| 3wk |  |  |  |  |  |  | − | 0.2036 | 0.5687 |
| 4wk |  |  |  |  |  |  |  | − | 0.2503 |
|  |  |  |  |  |  |  |  |  | − |

***Contra vs Sham***

| **Ca** | 1h | 1d | 2d | 3d | 1wk | 2wk | 3wk | 4wk | *Sham* |
| --- | --- | --- | --- | --- | --- | --- | --- | --- | --- |
| 1h | − | 0.5120 | 0.4139 | 0.1726 | 0.0229 | 0.8593 | 0.5162 | 0.3829 | 0.2959 |
| 1d |  | − | 0.7570 | 0.4225 | 0.0650 | 0.6421 | 0.1771 | 0.8252 | 0.7734 |
| 2d |  |  | − | 0.7412 | 0.2124 | 0.5054 | 0.1700 | 0.8834 | 0.8770 |
| 3d |  |  |  | − | 0.2641 | 0.2371 | 0.0433 | 0.5381 | 0.4630 |
| 1wk |  |  |  |  | − | 0.0343 | 0.0045 | 0.0877 | 0.0507 |
| 2wk |  |  |  |  |  | − | 0.4091 | 0.4983 | 0.4155 |
| 3wk |  |  |  |  |  |  | − | 0.1138 | 0.0602 |
| 4wk |  |  |  |  |  |  |  | − | 0.9882 |
|  |  |  |  |  |  |  |  |  | − |

***Contra vs Sham***

| **Fe** | 1h | 1d | 2d | 3d | 1wk | 2wk | 3wk | 4wk | *Sham* |
| --- | --- | --- | --- | --- | --- | --- | --- | --- | --- |
| 1h | − | 0.6241 | 0.3469 | 0.3536 | 0.9194 | 0.0595 | 0.6022 | 0.1001 | 0.7084 |
| 1d |  | − | 0.5422 | 0.5604 | 0.5079 | 0.0800 | 0.9419 | 0.1337 | 0.7900 |
| 2d |  |  | − | 0.9510 | 0.2615 | 0.2731 | 0.6127 | 0.4968 | 0.3547 |
| 3d |  |  |  | − | 0.2616 | 0.2298 | 0.6370 | 0.4254 | 0.3499 |
| 1wk |  |  |  |  | − | 0.0352 | 0.4952 | 0.0544 | 0.5686 |
| 2wk |  |  |  |  |  | − | 0.1096 | 0.5487 | 0.0306 |
| 3wk |  |  |  |  |  |  | − | 0.1936 | 0.7492 |
| 4wk |  |  |  |  |  |  |  | − | 0.0314 |
|  |  |  |  |  |  |  |  |  | − |

***Contra vs Sham***

| **Cu** | 1h | 1d | 2d | 3d | 1wk | 2wk | 3wk | 4wk | *Sham* |
| --- | --- | --- | --- | --- | --- | --- | --- | --- | --- |
| 1h | − | 0.1534 | 0.9078 | 0.1805 | 0.5063 | 0.1577 | 0.2462 | 0.0768 | 0.1192 |
| 1d |  | − | 0.1185 | 0.9435 | 0.4403 | 0.8800 | 0.8086 | 0.6493 | 0.9150 |
| 2d |  |  | − | 0.1416 | 0.4309 | 0.1251 | 0.1982 | 0.0573 | 0.0872 |
| 3d |  |  |  | − | 0.4910 | 0.8356 | 0.8656 | 0.6118 | 0.9910 |
| 1wk |  |  |  |  | − | 0.4138 | 0.6096 | 0.2479 | 0.4068 |
| 2wk |  |  |  |  |  | − | 0.7246 | 0.8126 | 0.7929 |
| 3wk |  |  |  |  |  |  | − | 0.5114 | 0.8391 |
| 4wk |  |  |  |  |  |  |  | − | 0.4902 |
|  |  |  |  |  |  |  |  |  | − |

***Contra vs Sham***

| **Zn** | 1h | 1d | 2d | 3d | 1wk | 2wk | 3wk | 4wk | *Sham* |
| --- | --- | --- | --- | --- | --- | --- | --- | --- | --- |
| 1h | − | 0.7823 | 0.0420 | 0.1737 | 0.3770 | 0.5384 | 0.5404 | 0.6436 | 0.8655 |
| 1d |  | − | 0.0543 | 0.2324 | 0.2303 | 0.6884 | 0.3496 | 0.8379 | 0.5631 |
| 2d |  |  | − | 0.3378 | 0.0075 | 0.1543 | 0.0114 | 0.0763 | 0.0106 |
| 3d |  |  |  | − | 0.0304 | 0.5192 | 0.0478 | 0.3171 | 0.0455 |
| 1wk |  |  |  |  | − | 0.1575 | 0.7581 | 0.1705 | 0.3384 |
| 2wk |  |  |  |  |  | − | 0.2351 | 0.8206 | 0.3547 |
| 3wk |  |  |  |  |  |  | − | 0.2628 | 0.5283 |
| 4wk |  |  |  |  |  |  |  | − | 0.4030 |
|  |  |  |  |  |  |  |  |  | − |
